# Mertk-expressing microglia influence oligodendrogenesis and myelin modelling in the CNS

**DOI:** 10.1186/s12974-023-02921-8

**Published:** 2023-11-06

**Authors:** Linda T. Nguyen, Andrea Aprico, Eze Nwoke, Alexander D. Walsh, Farrah Blades, Raphael Avneri, Elodie Martin, Bernard Zalc, Trevor J. Kilpatrick, Michele D. Binder

**Affiliations:** 1 The Florey Institute of Neuroscience and Mental Health, Parkville, Melbourne, Australia; 2 Inserm, CNRS, Institut du Cerveau, AP-HP Pitié-Salpêtrière Hospital, Sorbonne Université, Paris, France; 3 Present Address: Department of Anatomy and Physiology, University of Melbourne, Parkville, Melbourne, Australia; 4 Present Address: Crux Biolabs, Bayswater, VIC 3153 Australia; 5 Present Address: Cognitive Neuroepigenetics Laboratory, Queensland Brain Institute, The University of Queensland, Brisbane, QLD Australia; 6 Present Address: Centre for Solar Biotechnology, Institute for Molecular Biosciences, University of Queensland, St Lucia, Brisbane, Australia; 7 Present Address: Department of Molecular Biology, Ariel University, 40700 Ariel, Israel

## Abstract

**Background:**

Microglia, an immune cell found exclusively within the CNS, initially develop from haematopoietic stem cell precursors in the yolk sac and colonise all regions of the CNS early in development. Microglia have been demonstrated to play an important role in the development of oligodendrocytes, the myelin producing cells in the CNS, as well as in myelination. Mertk is a receptor expressed on microglia that mediates immunoregulatory functions, including myelin efferocytosis.

**Findings:**

Here we demonstrate an unexpected role for Mertk-expressing microglia in both oligodendrogenesis and myelination. The selective depletion of Mertk from microglia resulted in reduced oligodendrocyte production in early development and the generation of pathological myelin. During demyelination, mice deficient in microglial Mertk had thinner myelin and showed signs of impaired OPC differentiation. We established that Mertk signalling inhibition impairs oligodendrocyte repopulation in *Xenopus* tadpoles following demyelination.

**Conclusion:**

These data highlight the importance of microglia in myelination and are the first to identify Mertk as a regulator of oligodendrogenesis and myelin ultrastructure.

**Supplementary Information:**

The online version contains supplementary material available at 10.1186/s12974-023-02921-8.

## Background

Multiple sclerosis (MS) is an inflammatory demyelinating disease of the central nervous system (CNS), and is a common cause of neurological disability in young adults. The majority of current MS therapies treat the inflammation and reduce clinical activity of the early stages of MS. However, in the long term most people with MS develop progressive disability. An alternative therapeutic approach to treat MS is to identify new targets for directly influencing demyelination, thereby promoting functional recovery and limiting the propensity for otherwise demyelinated axons to degenerate. Recent discoveries have demonstrated that microglia play an important role in the development of oligodendrocytes, the myelin producing cells in the CNS, as well as in myelination [1–3].

Microglia are the resident immune cells of the CNS. In addition to immune regulation, microglia contribute to homeostasis in the brain and are also known for their involvement in sculpting neuronal circuitry in the developing brain by engulfing synaptic spines [4, 5] and neuronal cell bodies [6]. Microglia have been shown to regulate oligodendrogenesis by secreting growth factors and cytokines to promote oligodendrocyte progenitor cell (OPC) maturation [2, 7]. Researchers have also observed microglia making contact with, and engulfing myelin sheaths in zebrafish larvae, suggesting that these cells take part in refining aberrantly formed myelin [8, 9].

Mertk is a member of the TAM (Tyro3, Axl, Mertk) family of receptor tyrosine kinases, which can be activated by two ligands, growth arrest specific gene 6 (Gas6) and Protein S (Pros1) [10]. Mertk is involved in mediating key microglial and macrophage functions including phagocytosis of apoptotic cells and myelin debris [11–13] and regulating expression of anti-inflammatory markers [14, 15]. In addition, variations in the *MERTK* gene have been associated with increased MS susceptibility [16] as well as an increased likelihood of developing secondary progressive MS [17].

In the CNS, Mertk is predominantly expressed by microglia [18, 19]. Multiple lines of evidence using knockout mice have identified a central role for TAM receptor signalling in modifying the outcome of pre-clinical models of demyelination and remyelination [20–24], although all have been undertaken in adult mice with complete deletion of either the receptors or ligands, leaving a substantial gap in knowledge in respect to understanding the developmental and cell-specific roles that Mertk may play in myelination.

In order to understand the cell-specific role of Mertk in microglia, we developed a mouse model in which deletion of Mertk is driven by the Cx3Cr1 promotor, which in the central nervous system is confined to microglia. The loss of Mertk in microglia resulted in evidence of mature myelin which was prone to splitting. During development we observed decreased proliferation in the corpus callosum, and fewer newly generated oligodendrocytes. This developmental alteration in oligodendrocyte production dynamics was accompanied by disrupted myelin gene expression. In the cuprizone model of demyelination, Mertk cKO mice experienced a worsened course of demyelination compared with Mertk WT mice, whereby myelin was significantly thinner, a deficit which continued through the earliest stages of remyelination in this model. We expanded this work to a transgenic *Xenopus laevis* model of demyelination and observed for the first time that the promyelinating effect of TAM signalling was conserved in *Xenopus*, and that Mertk inhibition is also detrimental to oligodendrocyte repopulation in this model. We have thus identified a previously unsuspected role for Mertk^+ve^ microglia in developmental myelination, along with establishing the importance of microglial Mertk in demyelination remyelination in the CNS.

## Results

### Mertk is efficiently deleted from microglia in the presence of the Cx3Cr1 cre driver

**Fig. 1:**
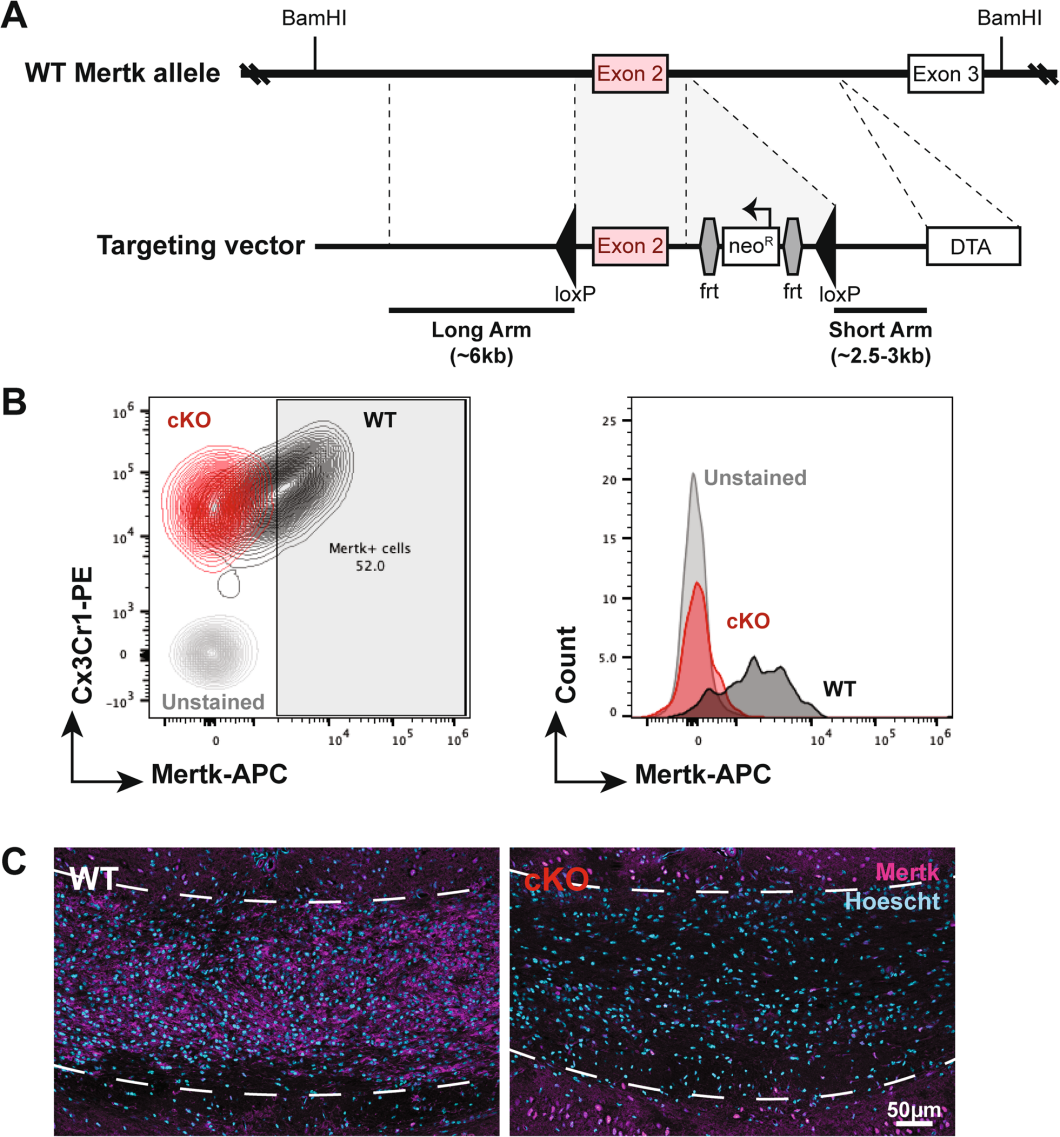
Mertk is efficiently deleted from microglia in the presence of the Cx3Cr1 cre driver **A** Floxed Mertk mice were generated by the introduction of loxP sites on either side of exon 2 of the Mertk gene. An frt-flanked pGK-neomycin (neo^R^) cassette was also introduced for positive selection of embryonic stem cells. A diphtheria toxin A (DTA) cassette was introduced to allow for negative selection. **B** Representative flow cytometry plot (left) and corresponding histogram (right), indicating Mertk expression in Cx3Cr1^+ve^ microglia generated from Mertk WT mice (black) compared with Cx3Cr1^+ve^ microglia derived from Mertk cKO (red). **C** Widespread Mertk immunopositivity (magenta) is observed in the corpus callosum (dashed lines) of Mertk WT mice following 5 weeks of cuprizone-challenge to induce Mertk expression. Conversely, little to no Mertk expression is observed in the corpus callosum of Mertk cKO mice. Hoechst-labelled nuclei in blue. Scale bar represents 50 µm

To assess the role of Mertk in microglia, we developed a novel conditional deletion model. A Mertk-*floxed* mouse line was developed in which loxP sites were introduced upstream and downstream of exon 2 of Mertk (Fig. 1A). Recombination of the loxP sites in the presence of cre results in early stop codons in the Mertk transcript. We used the previously developed Cx3Cr1 cre mouse line [25] to drive recombination in Cx3Cr1^+ve^ cells, including microglia within the CNS. We first assessed the efficiency of deletion in microglia by generating mixed glial cultures from Mertk^fl/fl^ Cx3Cr1^cre/wt^ (Mertk cKO) and Mertk^fl/fl^ Cx3Cr1^wt/wt^ (Mertk WT) littermate control brain tissue. Cx3Cr1^+ve^ microglia from Mertk cKO cultures were devoid of Mertk expression, as assessed by flow cytometry. In comparison, Mertk was detected in 50–60% of Cx3Cr1^+ve^ cells in mixed glial cultures derived from WT brains (Fig. 1B). As adult microglia do not significantly express Mertk in the steady state, we assessed Mertk expression in tissue from animals challenged with the toxin cuprizone, which induces microglial activity and upregulates Mertk expression [20]. As expected, tissue from WT mice showed strong expression of Mertk throughout the corpus callosum (Fig. 1C). In contrast, there was little to no Mertk expression observed in cKO tissue (Fig. 1C).

### Pathological myelin is observed in Mertk cKO mice independent of changes in cell density

**Fig. 2:**
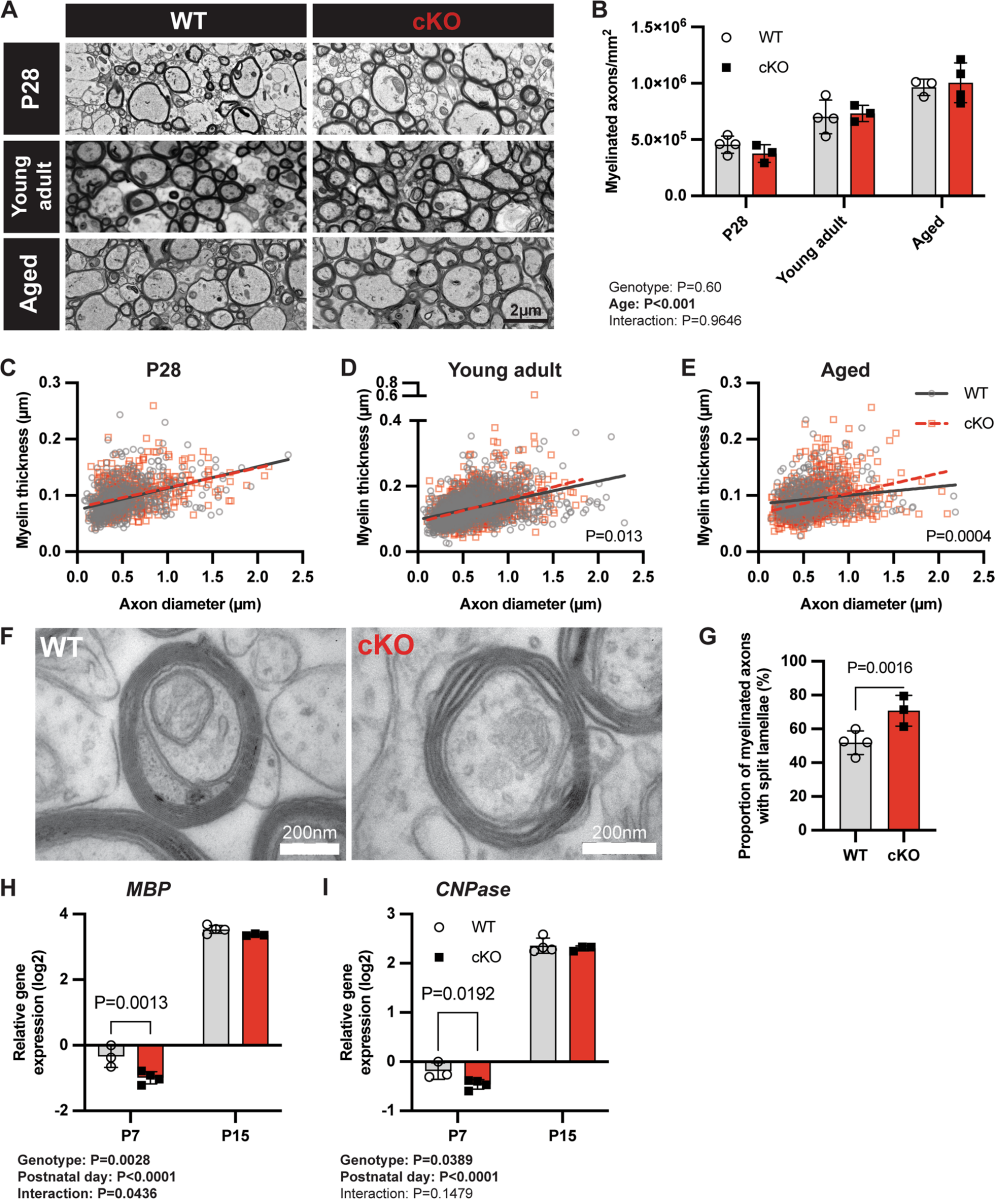
Loss of microglial Mertk is associated with pathological myelin and reduced myelin gene expression. **A** Representative TEM images of myelinated axons in the corpus callosum of Mertk WT and cKO from P28, young adult (8–12 weeks) and aged (12 months) mice. **B** The density of myelinated axons was similar between WT and Mertk cKO mice at all time-points (two-way ANOVA; P > 0.05). Plots of myelin thickness versus axon calibre for P28 (**C**), adult (**D**) and aged (**E**) mice. A moderate shift towards thicker myelin was observed in adult (linear regression; *P = *0.013) and aged (linear regression; *P = *0.0004) mice was observed. **F** Representative high power TEM images of a myelinated axon in Mertk WT and cKO mice, demonstrating split lamellae in the latter. G Numbers of myelinated axons with at least one split lamellae are significantly increased in Mertk cKO mice (Fisher's exact test; *P = *0.0016). The expression of the genes encoding MBP (H) and CNPase (**I**) is significantly reduced at P7 in the corpus callosum of Mertk cKO mice compared with WT, although this normalises by P15 (Student's t-test). Data represent mean ± SD with *n = *3–5 biological replicates per genotype. For myelin thickness, all myelinated axons in a minimum of 6 non-overlapping images were measured. Data represent mean ± SD

As a prelude to assessing the role of Mertk^+ve^ microglia in demyelination, we first assessed the effect of conditional deletion of Mertk upon myelination in the unchallenged mouse brain. We therefore assessed myelin density and morphology at P28, young adult (8–12 weeks) and aged mice (12 months) (Fig. 2A). The density of myelinated axons was similar between Mertk WT and cKO at all ages examined (Fig. 2B). Morphometric analysis of myelinated fibres showed no difference in myelin thickness at P28 (Fig. 2C), but did identify a subtle increase in myelin thickness in both young adult (Fig. 2D) and aged mice (Fig. 2E). Using high power TEM to assess myelin in adult Mertk WT and cKO mice we identified signs of myelin pathology, specifically lamellae splitting, in the absence of Mertk in microglia (Fig. 2F). Quantification of the number of axons with identifiable instances of split lamellae revealed a statistically significant increase of ~ 20% in the number of split lamellae in Mertk cKO mice (Fig. 2G). The increase in the number of split lamellae likely accounts for the apparent increase in myelin thickness observed in young adult and aged mice.

Splitting of lamellae can arise as a result of a failure of myelin compaction. Myelin proteins such as myelin basic protein (MBP) and 2',3'-cyclic-nucleotide 3'-phosphodiesterase (CNPase) are critical in the formation of compact myelin during development [26]. We therefore assessed expression of these myelin genes during early postnatal development in the corpus callosum of Mertk WT and cKO mice. We observed a significant reduction in the expression of MBP (~ threefold) and CNPase (~ twofold) in the corpus callosum of Mertk cKO mice at P7, although expression had normalised by P15 (Fig. 2H, I).

To determine if the change in myelin morphology was a consequence of altered inflammation or oligodendrocyte maturation, we performed immunostaining to assess the density of microglia as well as oligodendrocyte lineage cells (Additional file 1: Fig. S1). The increase in pathological myelin observed in the Mertk cKO mice was not accompanied by any alteration in the densities of either Iba1^+ve^ microglia, Olig2^+ve^/PDGFRα^+ve^ OPCs or CC1^+ve^ oligodendrocytes, indicating that the myelin aberrations are not a direct result of loss of oligodendrocytes or changes in the density of microglia.

### The loss of microglial Mertk results in altered proliferation in the corpus callosum of postnatal mice

**Fig. 3:**
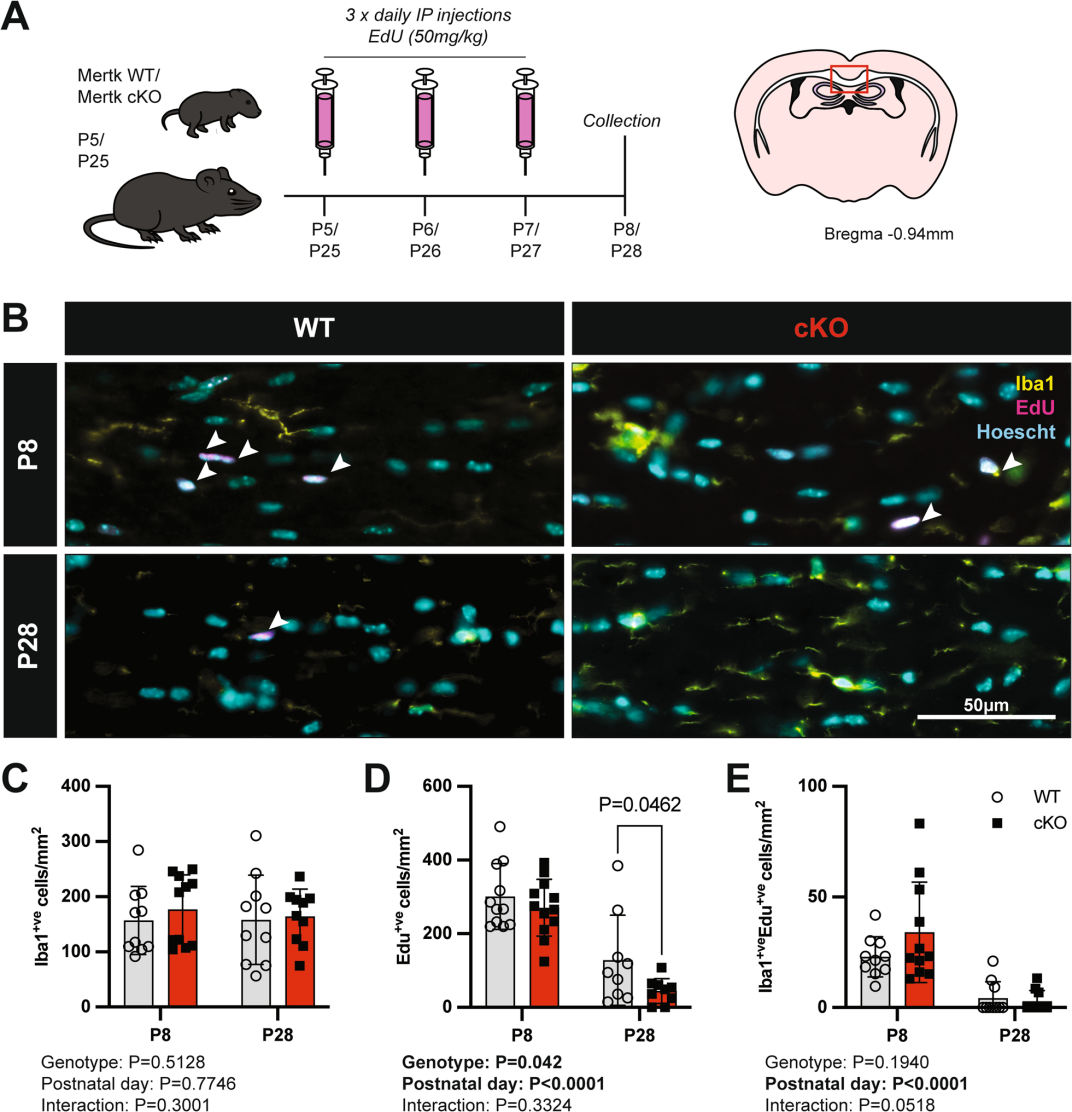
Early postnatal EdU incorporation is reduced in Mertk cKO animals, independent of changes in microglial density or proliferation. **A** EdU-labelling paradigm. Mertk WT or Mertk cKO mice aged P5 and P25 were injected with EdU (50 mg/kg) over three consecutive days prior to collection for immunohistochemistry (IHC) analysis at the midline of the corpus callosum at P8 and P28, respectively (n = 9–12 mice per group). **B** Representative immunofluorescence images of P8 Mertk WT and cKO corpus callosum tissue stained for Iba1 (yellow) and EdU (magenta), in addition to Hoechst nuclear stain (cyan). Arrowheads indicate EdU^+ve^/Iba1^+ve^ cells. Scale bar corresponds to 50 µm. **C** The density of Iba1^+ve^ microglia is similar between genotypes. **D** Fewer EdU^+ve^ cells/mm^2^ were observed in the corpus callosum of Mertk cKO mice (*P = *0.042), with a significant reduction in Edu^+ve^ cells at P28 (*P = *0.0462). **E** Microglial proliferation was not altered in the absence of Mertk. Data represent mean ± SD. Statistical significance determined using 2-way ANOVA with Fisher’s LSD

In light of the changes to myelin ultrastructure which were not explained by any alteration in the density of microglia or myelinating cells in the adult, as well as the developmental alteration in myelin gene expression, we next explored whether these changes were associated with any alterations in the generation or proliferation of glial cells in the early postnatal brain. We therefore administered the thymidine analogue 5-ethynyl-2’-deoxyuridine (EdU) to both Mertk cKO and WT mice for 3 days prior to collection of mouse brains at both P8 and P28 (Fig. 3A). We then used immunofluorescent staining to identify Iba1^+ve^ microglia in the corpus callosum at postnatal (P) day 8 and at P28 (Fig. 3B). The density of Iba1^+ve^ microglia was similar between Mertk WT and Mertk cKO at both ages examined (Fig. 3C). Although the loss of Mertk did not appear to influence total microglial densities, there was a significant effect of Mertk-deficiency on the density of EdU^+ve^ cells overall (Fig. 3D). There was an approximately 10% reduction in EdU incorporation in Mertk cKO mice compared with WT at P8, increasing to a 35% reduction in EdU^ +ve^ cells in Mertk cKO mice at P28 (Fig. 3D), indicating reduced cell proliferation in the absence of microglial Mertk. Interestingly, this reduction in proliferation was not reflected by any alteration in the densities of Iba1^+ve^/EdU^+ve^ newly generated microglia at either P8 or P28 (Fig. 3E).

### Oligodendrocyte production and progenitor proliferation are reduced in postnatal mice in the absence of Mertk^+ve^ microglia

**Fig. 4:**
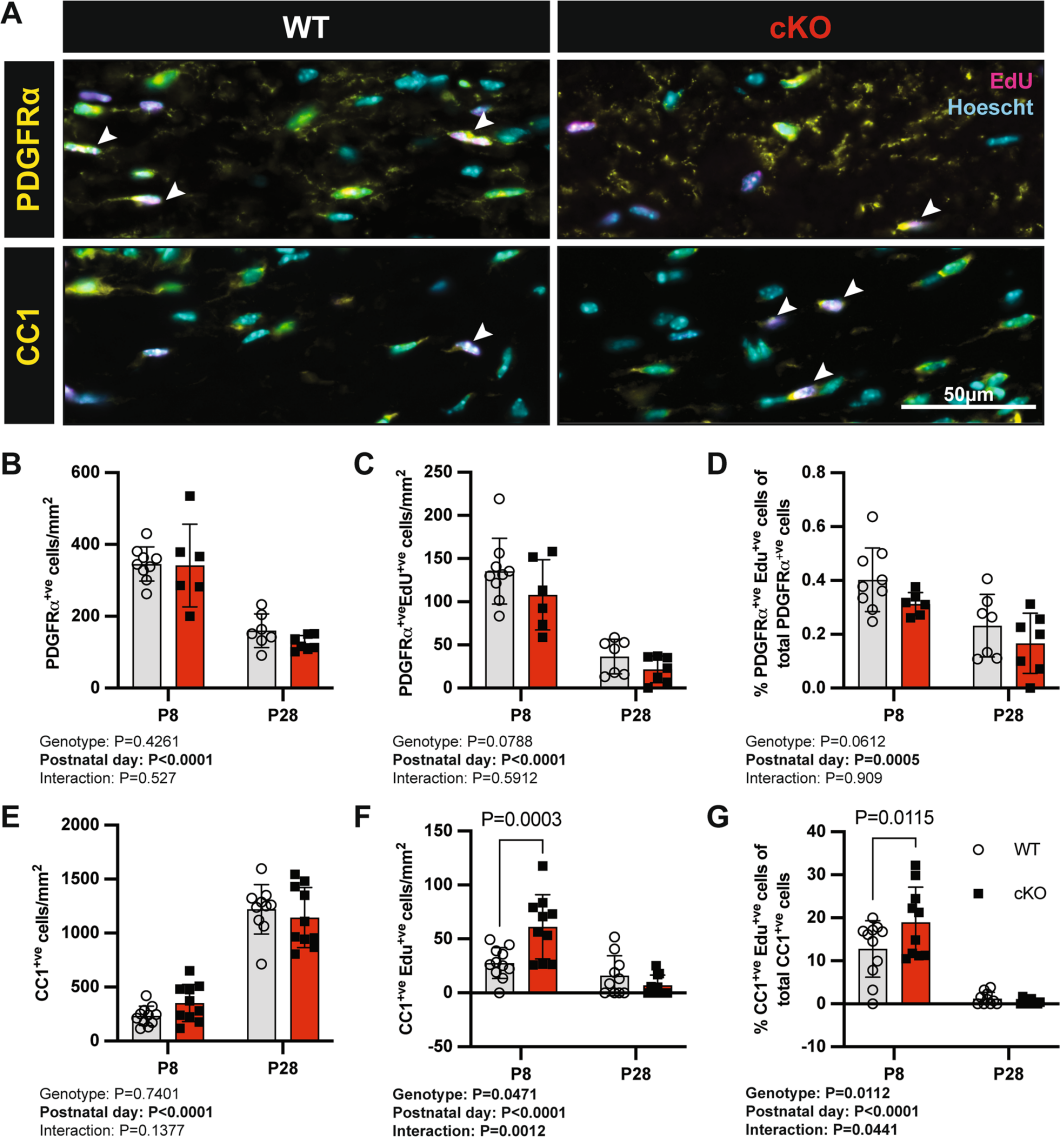
Altered mature oligodendrocyte production in the absence of microglial Mertk during development. **A** Representative immunofluorescence images of P28 Mertk WT (left) and cKO (right) corpus callosum tissue stained for the OPC marker PDGFRα and mature oligodendrocyte marker CC1 (yellow), EdU (magenta) in addition to nuclear Hoechst stain (cyan). Arrowhead indicates EdU^+^/CC1^+^ cell. Scale bar corresponds to 50 µm. **B** The density of PDGFRα^+ve^ OPCs was similar between genotypes. **C** A trend towards a reduction in the density of proliferating (Edu^+ve^) PDGFRa^+ve^ OPCs was observed as well as a trend towards a reduction in the proportion of proliferating OPCs (**D**). **E** The density of CC1^+ve^ cells/mm^2^ was not different between genotypes; however, the density of newly generated mature oligodendrocytes (CC1^+ve^ Edu^+ve^) was increase twofold at P8 (**F**). **G** Similarly, the proportion of newly generated CC1^+^ve mature oligodendrocytes was increased at P8. Data represent mean ± SD. Statistical significance determined using 2-way ANOVA with Fisher’s LSD

As the reduction in EdU incorporation observed in the corpus callosum could not be attributed to a reduction in microglial proliferation, we explored the proliferation of other cell types. We focussed on the OPC, which is the predominant proliferating cell in the postnatal corpus callosum, and used immunostaining to assess the density and proliferation of PDGFRα^+ve^ OPCs (Fig. 4A). The density of PDGFRα^+ve^ cells was similar between WT and cKO mice (Fig. 4B). In contrast, we observed a trend towards a reduction in the density of PDGFRα^+ve^/EdU + ve cells in Mertk cKO mice (~ 20% reduction in Mertk cKO at P8; ~ 40% at P28, Fig. 4C), as well as the proportion of dividing PDGFRα^+ve^ cells (Fig. 4D). To determine if this reduction in OPC proliferation was coupled with altered oligodendrocyte generation, we used immunofluorescence to determine the density of CC1^+ve^ mature OLs, in combination with EdU (Fig. 4A). The overall density of CC1^+ve^ oligodendrocytes was similar between Mertk WT and cKO mice (Fig. 4E). However, the dynamics of oligodendrocyte generation appeared altered, with a twofold increase in the density of CC1^+ve^EdU^+ve^ cells at P8 (Fig. 4F). Altered oligodendrogenesis in Mertk cKO mice was consistent with the changes in the density of newly generated oligodendrocytes, which proportion was increased at P8 (Fig. 4G).

We next wished to rule out the possibility that the alteration in OPC proliferation we observed in vivo was a direct result of loss of Mertk in OPCs, given that a subset of OPCs have recently been reported to express Cx3Cr1 [27]. We first used flow cytometry to assess Cx3Cr1 and Mertk expression in OPCs. We observed a subset of OPCs that were also Cx3Cr1^+ve^ (< 10%), but there was no detectable Mertk in these cells (Additional file 2: Fig. S2). We also established monocultures of OPCs purified from Mertk WT and Mertk cKO littermates. OPCs were cultured in the presence of the thymidine analogue bromodeoxyuridine (BrdU) to label proliferating cells. The proliferation of OPCs derived from Mertk cKO was similar to that of OPCs derived from Mertk WT mice, suggesting that the impairment in oligodendrocyte production in vivo is cell non-autonomous (Additional file 3: Fig. S3).

In light of the cell non-autonomous nature of the reduction in OPC proliferation in vitro, we undertook a candidate gene approach to determine if the loss of microglial Mertk impacts microglial expression of soluble factors that could affect OPC proliferation. We used qPCR to assess the expression of the TAM receptor ligand genes (*Gas6, Pros1*) as well as the known OPC proliferation factors *Fgf2, Pdgfra* and *Lif*. We also assessed the expression of the proliferation-region-associated microglia signature genes *Igf1, Lgals3, Spp1, Gpnmb* [28]. The expression of these genes was similar between microglia derived from Mertk WT mice and microglia derived from Mertk cKO mice (Additional file 3: Fig. S3), suggesting that these signalling pathways are not altered in cKO mice.

### Demyelination is worsened in the absence of microglial Mertk

**Fig. 5:**
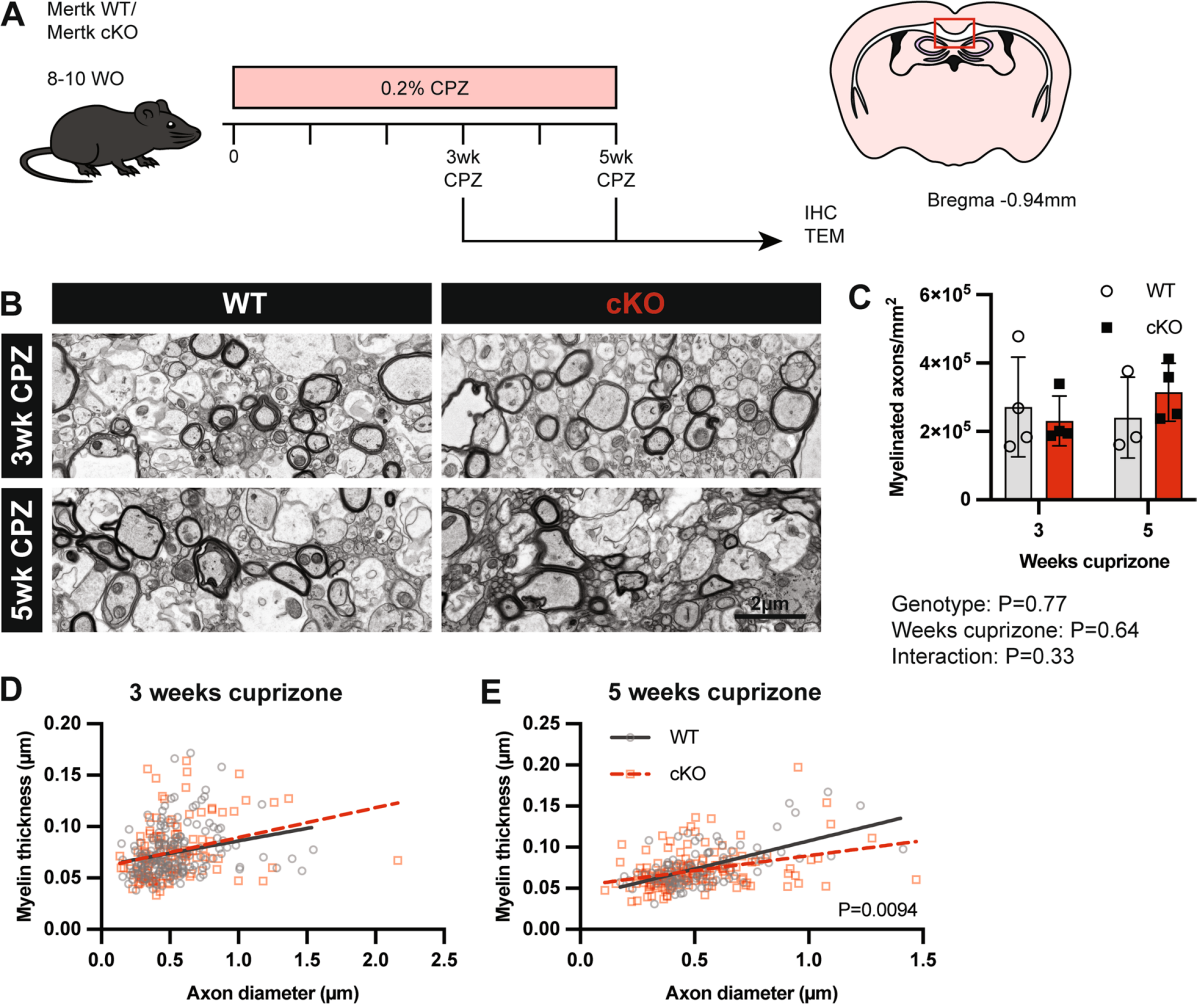
Demyelination is worsened in the absence of microglial Mertk. **A** Demyelination was induced in Mertk WT and cKO mice with oral administration of cuprizone for 3 or 5 weeks. **B** Representative TEM images of corpus callosum tissue from WT and cKO animals following 3 or 5 weeks cuprizone challenge. Scale bar represents 2 µm. **C** The density of myelinated axons was similar between Mertk WT and cKO mice following 3 and 5 weeks cuprizone-induced demyelination (2-way ANOVA; *P = *0.77). **D** Myelin thickness was similar between Mertk WT and cKO mice following 3 weeks cuprizone-induced demyelination (linear regression; *P* > 0.05). **E** At 5 weeks, myelin was significantly thinner in Mertk cKO mice compared with WT mice (linear regression; *P = *0.0094). All data obtained *n = *4 mice/group. Myelinated axon data represent means ± SD

We next asked whether the changes in the trajectory of oligodendrocyte development and adult myelin morphology would alter the outcome of central demyelination in the Mertk cKO mice. We induced demyelination using the toxin cuprizone (bis-cyclohexanone-oxaldihydrazone). Mice were administered chow containing 0.2% cuprizone (w/w) for up to 5 weeks (Fig. 5A). We used TEM to determine the density of myelinated axons in the corpus callosum following 3 and 5 weeks of cuprizone challenge (Fig. 5B). The density of myelinated axons was similar between Mertk WT and cKO mice following both 3 and 5 weeks of cuprizone-induced demyelination (Fig. 5C).

As we had previously identified alteration in myelin structure during development, we also assessed myelin thickness during the course of cuprizone-induced demyelination. In contrast to our observations in unchallenged adult mice, we did not observe any differences in myelin thickness following 3 weeks of demyelination in Mertk cKO mice compared with Mertk WT mice (Fig. 5D). Following 5 weeks of cuprizone challenge myelin in the corpus callosum of Mertk cKO mice was significantly thinner compared with Mertk WT, which was more apparent in larger calibre axons (diameter > 1 µm) (Fig. 5E).

**Fig. 6:**
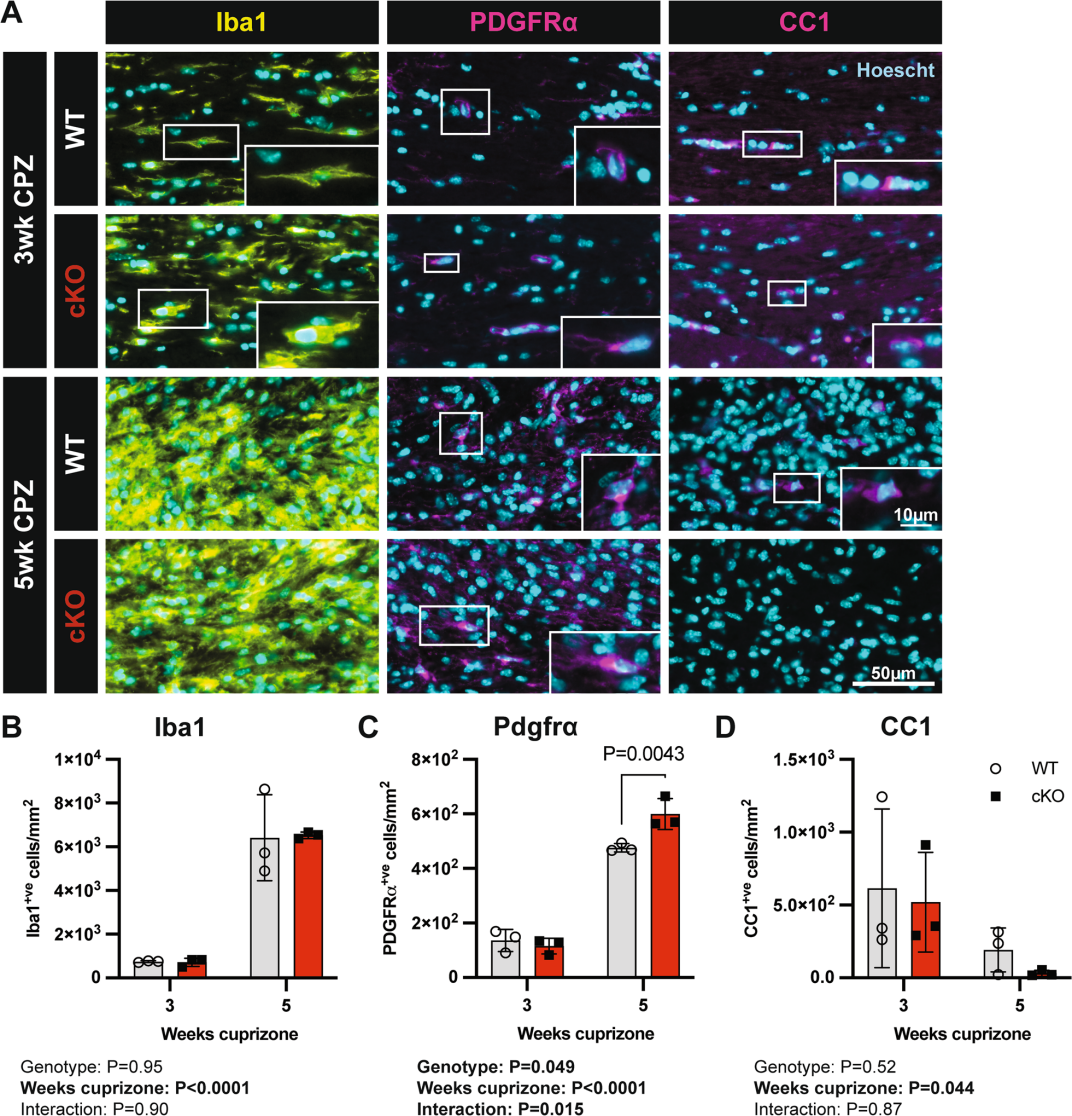
Mertk cKO animals display dysregulated OPC/OL dynamics during cuprizone-mediated demyelination. **A** Representative immunostaining of the corpus callosum from Mertk WT and cKO animals following 3 and 5 weeks cuprizone-challenge. Hoechst-labelled nuclei in cyan. Scale bar represents 50 µm. Inset scale bar represents 25 µm. **B** The density of Iba1^+ve^ microglia was similar between Mertk WT and cKO mice. **C** The density of PDGFRα^+ve^ OPCs was altered over the course of cuprizone-induced demyelination, with significantly more OPCs observed following 5 weeks demyelination. **D** A substantial reduction in CC1^+ve^ oligodendrocytes was observed in the absence of microglial Mertk, although this did not reach statistical significance. Data obtained from *n = *4 mice per group. Data represent means ± SD. Statistical significance was determined using two-way ANOVA with Fisher's LSD

To determine if inflammatory responses or oligodendrocyte development was altered during demyelination in the absence of microglial Mertk, we used immunohistochemical staining to assess glial cell densities in the corpus callosum (Fig. 6A). As expected, the density of microglia increased robustly over the course of cuprizone-induced demyelination, although the microglial response was similar between Mertk cKO and Mertk WT mice (Fig. 6B). In contrast, we observed a change in the density of PDGFRα^+ve^ OPCs in the corpus callosum of Mertk cKO mice compared with WT mice, with a 20% increase in the density of PDGFRα^+ve^ OPCs following 5 weeks of cuprizone-induced demyelination (Fig. 6C). This was accompanied by a ~ fourfold reduction in the mean density of CC1^+ve^ oligodendrocytes in Mertk cKO compared with Mertk WT mice, although this did not reach statistical significance (Fig. 6D). The increase in OPCs and reduction in mature oligodendrocytes could indicate that OPC differentiation is impaired in the absence of microglial Mertk.

### Microglia derived from Mertk cKO mice show impaired myelin efferocytosis

**Fig. 7:**
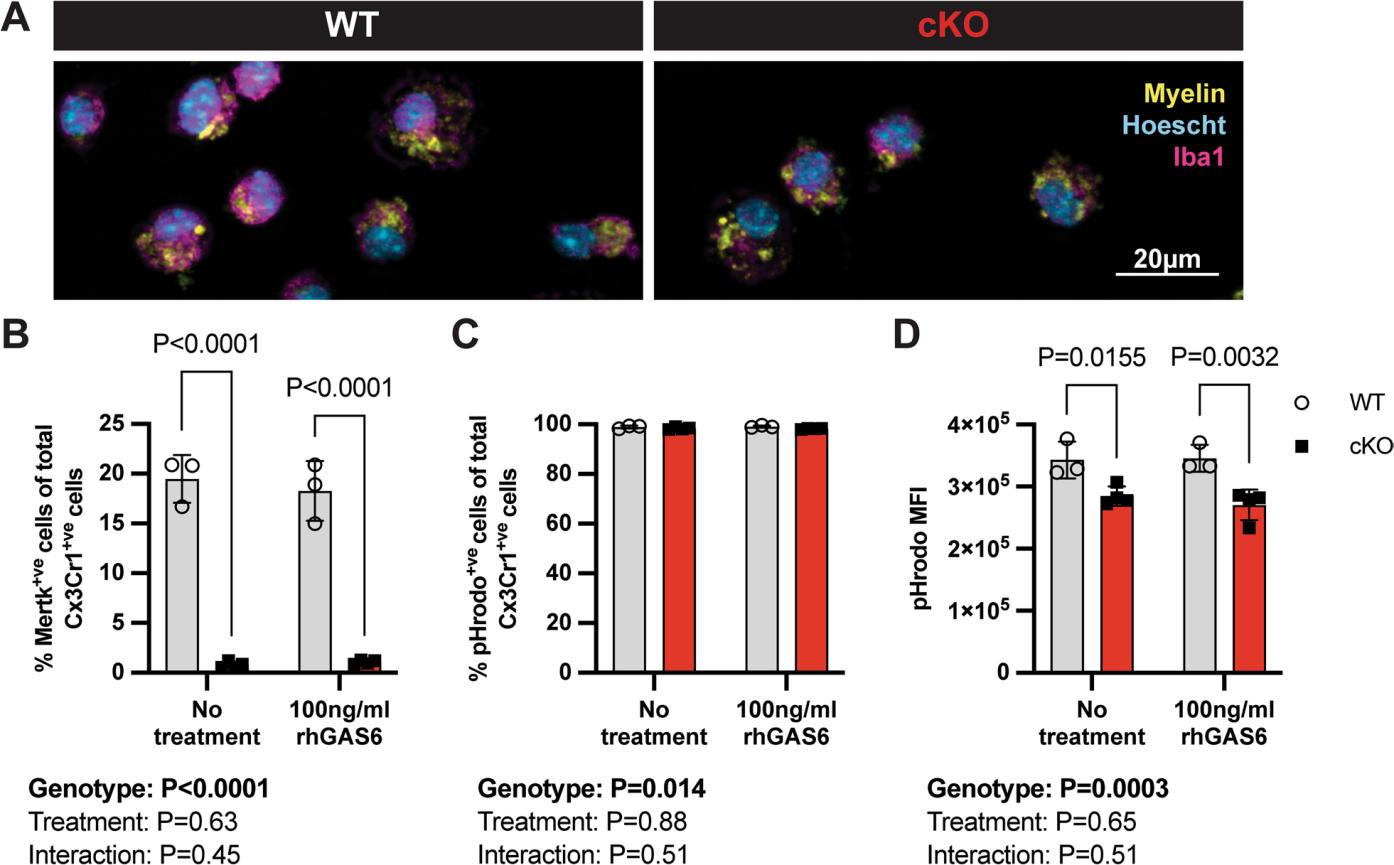
Myelin phagocytosis is impaired in Mertk cKO cells in vitro. **A** Representative immunofluorescence images of fluorescently labelled myelin (yellow) engulfed by Iba1^+ve^ microglia (magenta), generated from Mertk WT and cKO tissue. Hoechst-labelled nuclei in cyan. Scale bar represents 20 µm. **B** Cx3Cr1 + ve cKO microglia show almost complete deletion of Mertk (*P* < 0.0001). Expression of Mertk was not altered by treatment with rhGAS6 (*P* > 0.05). **C** Following delivery of pHrodo^+ve^ myelin to the cultures, the vast majority of Cx3Cr1^+ve^ cells were also pHrodo^+ve^, although a very small (< 1%) reduction in the percentage of pHrodo^+ve^ cells was observed in Mertk cKO cultures (*P = *0.014). No effect of rhGAS6 treatment on the proportion of pHrodo^+ve^ cells was observed (*P* > 0.05). D. Cx3Cr1^+ve^ Mertk cKO microglia engulfed significantly less myelin as determined using pHrodo median fluorescence intensity (MFI) (*P = *0.0003), irrespective of rhGAS6 treatment (two-way ANOVA *P* > 0.05), indicating impaired myelin phagocytosis in Mertk-deficient microglia. *n = *3–4 biological replicates. Data represent means ± SD. Statistical significance determined using two-way ANOVA with Fisher’s LSD

In the light of the apparent impairment of OPC maturation during cuprizone-induced demyelination, which can result from inefficient clearance of myelin debris, we next asked whether Mertk cKO microglia were deficient in efferocytosis of myelin. We assessed the efferocytic capacity of Mertk-deficient microglia using mixed glial cultures derived from either Mertk WT or cKO brains. We employed myelin labelled with pHrodo, a pH-sensitive dye which is intensely fluorescent upon internalisation (Fig. 7A). We first confirmed that Mertk was almost completely undetectable in Cx3Cr1^+ve^ cKO microglia; in comparison ~ 20% of WT Cx3Cr1^+ve^ cells were Mertk^+ve^ (Fig. 7B). Mertk expression in WT microglia was not altered following treatment with the Mertk ligand rhGAS6 (Fig. 7B). Following incubation with pHrodo-labelled myelin, the vast majority of Cx3Cr1^+ve^ microglia (98–99%) internalised a detectable amount of myelin. A very small but statistically significant reduction in the number (< 1%) of Mertk cKO microglia internalising some myelin was observed (Fig. 7C). Unsurprisingly, given the high overall efficiency of efferocytosis, treatment with rhGAS6 did not further enhance myelin internalisation (Fig. 7C). In contrast, Mertk cKO Cx3Cr1^+ve^ microglia engulfed significantly less myelin debris per cell compared with WT microglia, as assessed using mean fluorescence intensity (MFI) (Fig. 7D). The addition of rhGAS6 did not enhance efferocytosis by either Mertk WT or Mertk cKO microglia (Fig. 7D), indicating that Mertk ligand is expressed at sufficient levels in mixed glial cultures to facilitate bridging between receptor and myelin debris.

### The loss of microglial Mertk has a limited influence upon remyelination dynamics

**Fig. 8:**
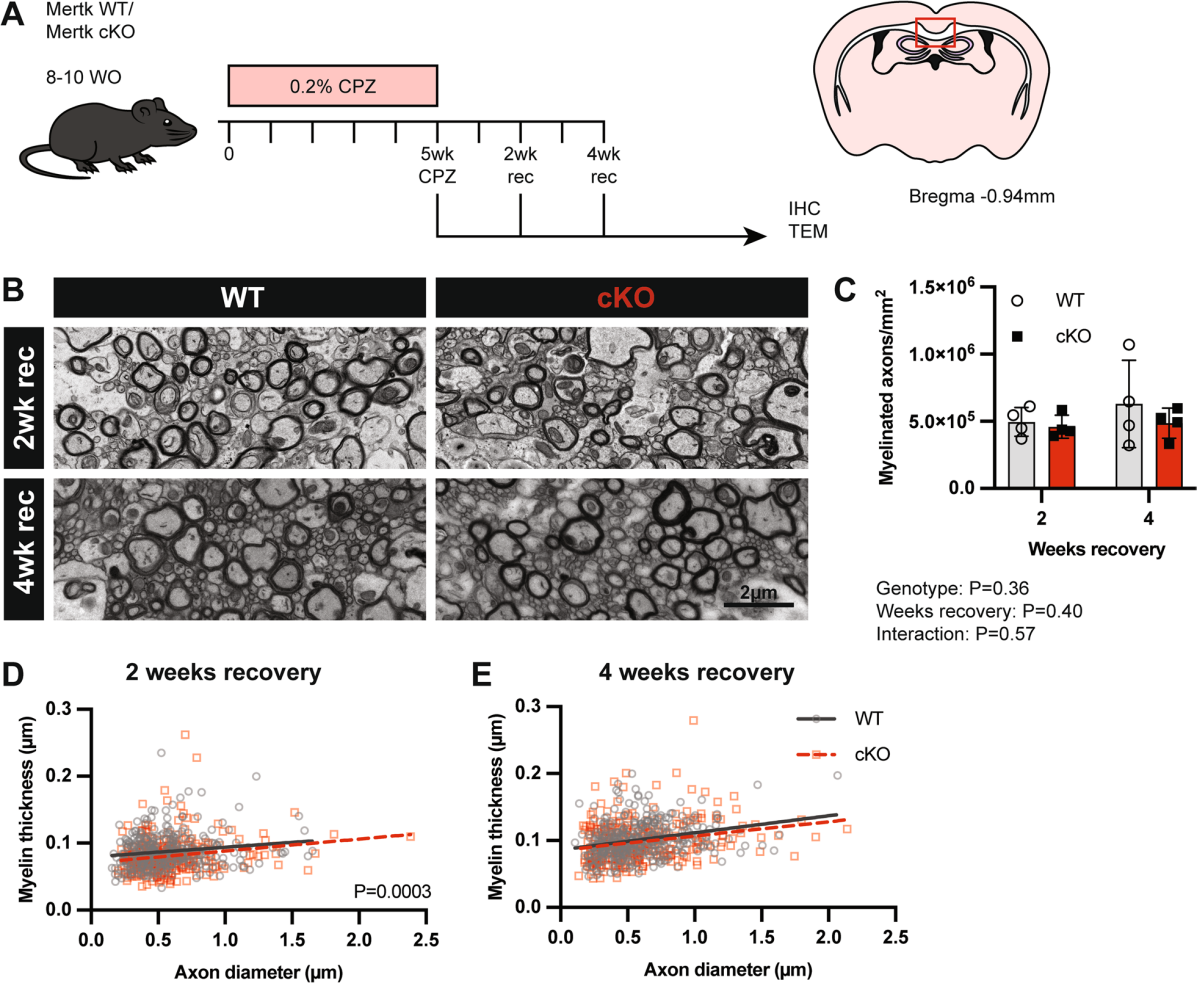
Remyelination is minimally delayed in Mertk cKO mice following cuprizone-challenge. **A** Demyelination was induced in Mertk WT and cKO mice with oral administration of cuprizone for 5 weeks followed by 2 or 4 weeks on normal feed to allow for remyelination to occur. **B** Representative TEM images of the corpus callosum of Mertk WT and cKO mice after 2 and 4 weeks recovery on standard chow. Scale bar represents 2 µm. **C** The density of myelinated axons was similar between Mertk WT and cKO mice following 2 and 4 weeks remyelination (2-way ANOVA; *P = *0.36). **D** Myelin thickness was significantly lower in Mertk cKO mice compared with Mertk WT following 2 weeks recovery (simple linear regression; *P = *0.0003), however by 4 weeks recovery (**D**), myelin thickness was similar between genotypes (simple linear regression; *P* > 0.05). Data obtained from *n = *4 mice per group. Myelin thickness analysed by simple linear regression. Myelinated axon data represent means ± SD

As phagocytosis of myelin debris by microglia is an important prerequisite to efficient remyelination following demyelination, we next wished to examine the effect of the loss of microglial Mertk upon this process. In order to assess the dynamics of myelin repair, we challenged Mertk WT and cKO mice with cuprizone for 5 weeks. Mice were then returned to a normal diet to allow endogenous remyelination to occur (Fig. 8A). We used TEM to assess the density of myelinated axons in the corpus callosum of mice following 2 and 4 weeks remyelination (Fig. 8B). Consistent with our findings during demyelination, axon density was similar between Mertk WT and cKO mice, indicating that the loss of microglial Mertk does not influence the initiation of remyelination in this model (Fig. 8C). Assessment of myelin thickness identified that myelin remained modestly but statistically significantly thinner following 2 weeks of recovery (Fig. 8D), consistent with our findings following 5 weeks of demyelination (Fig. 8E). By 4 weeks of recovery, however, myelin thickness was similar between Mertk WT and cKO mice (Fig. 8E).

We also assessed the cellular responses in the corpus callosum during remyelination using immunofluorescent staining (Additional file 4: Fig. S4). Although there was a trend towards an increase in the density of Iba^+ve^ microglia and PDGFRα^+ve^ cells, these changes were not accompanied by any alteration in the density of CC1^+ve^ oligodendrocytes. Taken together, these data indicate that deletion of Mertk from microglia has a limited effect upon the responses of microglia and oligodendrocyte lineage cells over the course of remyelination following cuprizone-induced demyelination.

Recent single-cell RNA sequencing data from full Mertk KO mice has suggested that a larger proportion of microglia in KO mice express a subset of IFNγ-related genes, which resulted in impaired remyelination [24]. We therefore assessed the expression of *Ifn*γ in our Mertk cKO model following cuprizone-induced demyelination and during recovery. However, we did not observe any differences in *Ifn*γ gene expression at any time point, suggesting that the phenotype we observe in the absence of microglial Mertk are independent of IFNγ (Additional file 5: Fig. S5).

### The beneficial effect of TAM receptor signalling is conserved in the Tg(mbp:GFP-NTR) Xenopus laevis tadpole model of demyelination

**Fig. 9:**
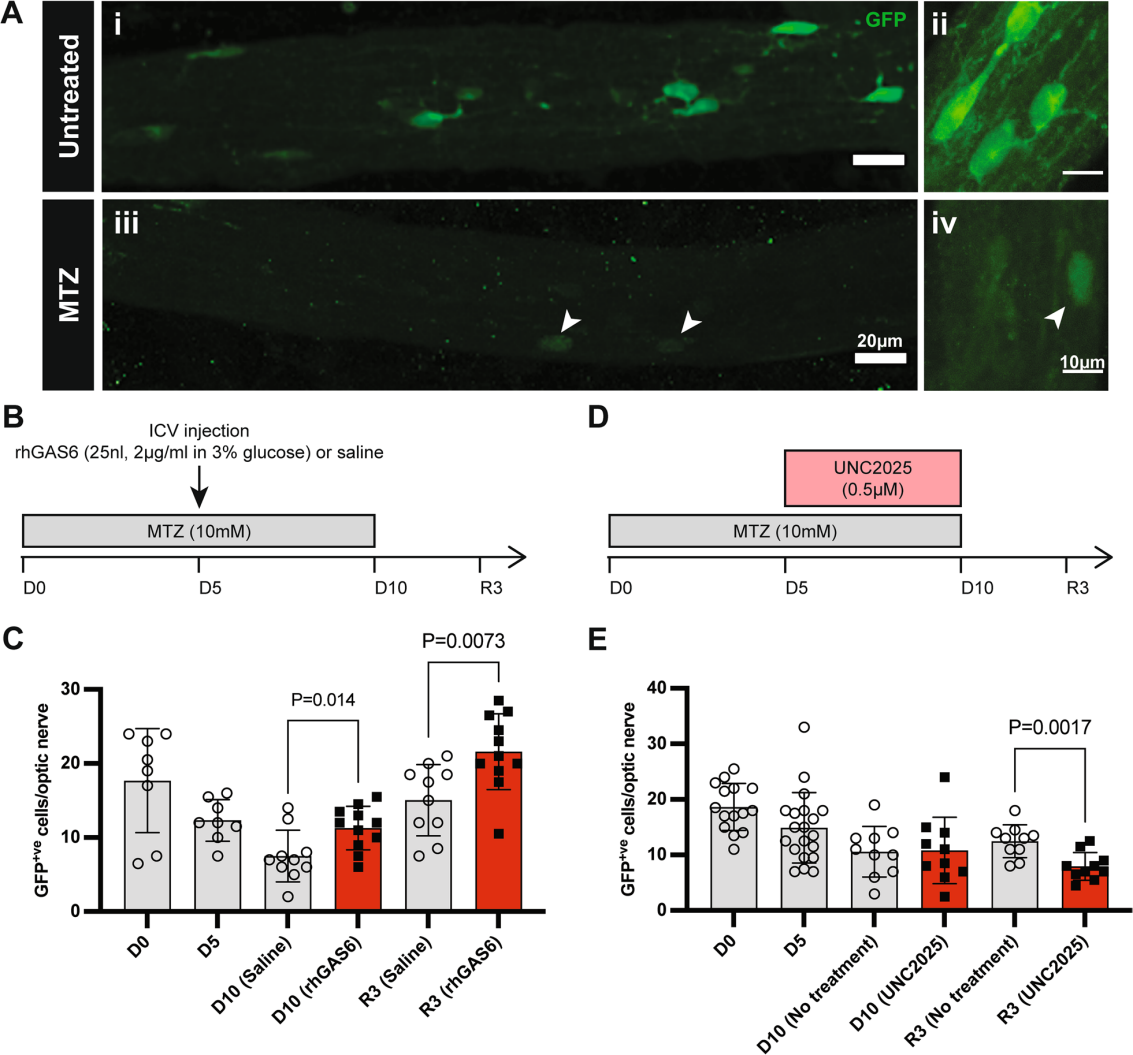
Remyelination in the *Tg*(*mbp:GFP-NTR) Xenopus laevis* tadpole model of demyelination is influenced by Mertk signalling. **A** Representative fluorescent images of GFP^+ve^ oligodendrocytes in the optic nerve of an untreated stage 50–55 *Tg*(*mbp:GFP-NTR) Xenopus laevis* tadpole at lower (i, iii) and higher (ii, iv) magnification. Following 10 days MTZ treatment, GFP^+ve^ cells are almost completely ablated. Arrowheads indicate MTZ-resistant oligodendrocytes. Left and right scale bars represent 20 µm and 10 µm, respectively. **B** Demyelination (**D**) was induced in stage 50–55 *Tg*(*mbp:GFP-NTR) Xenopus laevis* tadpoles by MTZ treatment in aquaria water between D0 and D10. Tadpoles were then returned to normal water to remyelinate (R) for 3 days. The number of GFP^+ve^ oligodendrocytes per optic nerve were counted on D0, D5, D10/11 and R3. To stimulate TAM signalling, tadpoles received an ICV injection of rhGAS6 (25nL volume, 2 µg/ml in 3% glucose) or vehicle on D5. **C** rhGAS6 treatment increased GFP^+ve^ cell numbers at D10 (*P = *0.014) and R3 (*P = *0.0073). D. To inhibit Mertk-signalling, tadpoles were treated with UNC2025 (0.5 µM) between D5 and D10 or given no treatment. **E** UNC2025 treatment significantly reduced the numbers of GFP^+^ cells in the optic nerves at R3. (*P = *0.0017). *n = *8–20 biological replicates, from 2 repeated experiments. Data represent means ± SD. Statistical significance was determined using unpaired *t-*tests

To complement our work in the microglial Mertk cKO mouse, we expanded our investigation of Mertk signalling to the transgenic *Tg*(*mbp:GFP-NTR) Xenopus laevis* tadpole model of demyelination [29]. In *Tg*(*mbp:GFP-NTR)* larvae, MBP-expressing oligodendrocytes in the CNS are fluorescently labelled with eGFP, allowing for live-imaging of individual oligodendrocytes (Fig. 9A). Loss of oligodendrocytes and subsequent demyelination can be induced by addition of the prodrug metronidazole (MTZ) into the aquaria water, which is converted by the nitroreductase (NTR) enzyme into a toxic product, resulting in cell death via apoptosis [29]. Treatment with MTZ (10 mM) for 10 days is sufficient to decrease by about 80% the number of myelin-forming oligodendrocytes from the optic nerves (Fig. 9A). If returned to standard aquarium water, tadpoles spontaneously remyelinate.

To determine if the promyelinating effect of TAM signalling was present in *Xenopus laevis*, we assessed the ability of exogenous rhGAS6 to enhance oligodendrocyte regeneration. *Tg*(*mbp:GFP-NTR)* tadpoles were treated with MTZ for 10 days, receiving an intracerebroventricular (ICV) injection of rhGAS6 or saline on demyelination day (D)5 (Fig. 9B). On D10, rhGAS6-treated tadpoles had significantly more GFP^+ve^ oligodendrocytes in their optic nerves compared with saline controls (Fig. 9C). This beneficial effect continued into the recovery phase of the experiment, as the optic nerves of rhGAS6-treated tadpoles contained significantly more GFP^+ve^ oligodendrocytes following 3 days recovery compared with saline controls (Fig. 9D). These results indicate that exogenous rhGAS6 treatment has a beneficial effect in demyelination and remyelination, and that the Gas6-TAM signalling axis is conserved in *Xenopus laevis*.

To determine if the beneficial effect of Gas6 signalling involved signalling via Mertk, we next employed the Mertk inhibitor UNC2025 during MTZ-induced demyelination. *Tg*(*mbp:GFP-NTR)* tadpoles were treated with MTZ for 10 days, and exposed to UNC2025 (0.5 µM) between D5 and D10 to assess the effects of Mertk inhibition on demyelination and remyelination (Fig. 9D). UNC2025 did not alter the number of GFP^+ve^ oligodendrocytes at D10, suggesting there is no role for Mertk in preventing oligodendrocyte death during demyelination. Conversely, UNC2025 did appear to inhibit GFP^+ve^ oligodendrocyte repopulation during recovery (Fig. 9D), suggesting a role for Mertk signalling during remyelination in *Xenopus laevis*.

## Discussion

Here, we have shown for the first time that deletion of microglial Mertk has negative impacts on both oligodendrogenesis and myelin ultrastructure. Using mice in which Mertk was conditionally deleted from microglia, we have investigated the role of microglial Mertk in myelination and identified impairments in oligodendrogenesis in cKO mice during development. These impairments were accompanied by changes in myelin gene expression and the identification of pathological myelin. These deficits were then amplified in the context of demyelination, as Mertk cKO animals showed signs of blocked OPC differentiation and thinner myelin after 5 weeks of cuprizone-challenge. Our work in the mouse is further supported by our findings in *Tg*(*mbp:GFP-NTR) Xenopus laevis* tadpoles, where pharmacological inhibition of Mertk also impaired oligodendrocyte production and/or maintenance.

Although Mertk was conditionally deleted from microglia, we did not observe any changes in microglial proliferation or densities throughout development. In our assessment of the Mertk cKO mouse, we observed a reduction in EdU incorporation in the corpus callosum during early development, which was not accounted for by reduced microglial proliferation. We determined that oligodendrocyte generation was dysregulated, suggesting that Mertk-expressing microglia interact with oligodendroglia to regulate cell numbers. Microglia have recently been shown to regulate the development of oligodendrocytes, and transcriptomic analysis has revealed gene signatures linked with proliferation-associated microglia (PAM) [28]. However, only a limited number of microglial factors have been specifically linked to perturbations in OPC proliferation and oligodendrocyte development, including insulin-like growth factor 1 (IGF1) [2] and neuropilin-1 (Nrp1) [30]. Our data unexpectedly add Mertk to the list of microglial-expressed factors which influence oligodendrogenesis, although we were unable to identify the molecular mechanism by which Mertk^+ve^ microglia promotes oligodendrocyte differentiation. We speculated that Mertk-deficient microglia would show reduced expression of one or more of the PAM signature genes associated with early OPC proliferation [28]. However, using a candidate approach we were unable to identify any gene expression changes in Mertk cKO microglia. It is possible that our candidate approach was insufficiently broad, and an untargeted method such as RNA sequencing is required.

Despite early developmental changes in OPC proliferation, the density of oligodendrocytes in Mertk cKO mice normalises by adulthood. Nonetheless, myelin in adult Mertk cKO animals was prone to lamellae splitting, to the point where corpus callosum myelin was significantly “thicker” than WT. Lamella splitting has been observed in both myelin mutants, such as in Plp KO mice [31], but also in disease models of demyelination, including the injection of anti-MOG antibodies into mice [32]. More generically, “loosening” of the myelin wraps is commonly seen as a consequence of aging [33], secondary degeneration following axonal injury [34] and myelin protein-deficiency [35, 36]. McNamara et al. (2023) recently showed that genetic ablation of microglia resulted in dysregulation of myelin formation, including a higher proportion of myelin outfoldings and increased myelin thickness [3]. Overall, the myelin pathologies observed in microglia-deficient mice are similar to the pathological myelin observed in the absence of Mertk^+ve^ microglia, such as apparent increases in myelin thickness.

While the exact reason for the pathological myelin phenotype in Mertk cKO mice cannot be explained from this dataset alone, the reduced level of MBP and CNPase gene expression at P7 may account for the splitting of myelin wraps. Conversely, and perhaps contradictorily, at the same developmental stage we observed an increase in the number of newly generated CC1^+ve^ oligodendrocytes. In other contexts, such as the loss of the transcription factor Hes5, premature differentiation of OPCs resulted in increased myelin gene expression [37]. In contrast, premature differentiation of OPCs in the absence of Id4, resulted in bidirectional changes in expression of specific myelin genes, with some increased in expression whilst other decreased [38]. Furthermore, the premature differentiation of OPCs only resulted in a modest and transient increase in the total number of CC1^+ve^ oligodendrocytes [38]. Nonetheless, although the molecular mechanism remains to be determined, our data are consistent with the hypothesis that Mertk^+ve^ microglia are the source of a factor that prevents premature differentiation of OPCs during early development. The identification of this factor in future studies is likely to represent an important step forward in understanding the mechanism by which microglia influence oligodendrocyte development.

In contrast, when subjected to the cuprizone-induced demyelination, Mertk cKO mice showed an increase in the density of OPCs. The increase in OPC density, which was accompanied by reduction in oligodendrocyte generation, could reflect two possible sequences of events. OPC maturation may be impaired and oligodendrocyte numbers fail to replenish during/following demyelination, perhaps as a result of deficient myelin clearance caused by microglial Mertk deletion. Alternatively, due to exacerbated, ongoing demyelination, oligodendrocytes in Mertk cKO mice could be particularly vulnerable, and hence die at a much higher rate than in WT mice. As a result, surviving OPCs proliferate to restore the oligodendrocyte population. Resolving this question will require further studies specifically assessing the proliferation of OPCs during cuprizone-induced demyelination.

A somewhat surprising result in our study was the limited effect of Mertk deletion upon the course of recovery from cuprizone-induced demyelination. This is in contrast to a similar study utilising complete developmental deletion of Mertk, which resulted in a significant delay in recovery in the density of myelinated axons following cuprizone-induced demyelination [24]. TAM receptors, including Mertk, have been shown to be expressed on NSCs [39]. As oligodendrocyte lineage cells are generated from NSCs, deletion in these cells may result in disturbances to normal OPC/OL generation. This is of particular relevance to the cuprizone model, in which OPCs derived from SVZ NSCs migrate to the corpus callosum, to replenish the oligodendrocyte population [40–42]. It has also been suggested that SVZ-derived NSCs can modulate microglial activity during demyelination by stimulating them to engulf myelin debris [43]. Furthermore, Mertk is expressed on endothelial cells, playing a role in maintaining BBB integrity [44] and signalling NSCs to commit to the oligodendrocyte lineage [45]. The differences between our results using microglial specific deletion of Mertk and that observed in the context of full deletion strongly suggest a broader role for Mertk in remyelination than simply phagocytosis of myelin debris. This may extend to non-CNS derived macrophages which may play a role in cuprizone-derived macrophages, and may be more extensively or efficiently deleted in the complete Mertk KO employed in the previously reported study [24].

Our experiments in *Xenopus laevis* tadpoles are also the first to show that TAM receptor signalling pathways are conserved in this species. Exogenous delivery of rhGAS6 into *Tg*(*mbp:GFP-NTR)* tadpoles during demyelination recapitulated the beneficial effects of rhGAS6 infusion seen in mouse models of demyelination [46, 47]. Delivery of rhGAS6 into the brains of *Tg*(*mbp:GFP-NTR)* tadpoles promoted oligodendrocyte repopulation following MTZ-induced demyelination. In addition, rhGAS6 injection also provided a protective effect against oligodendrocyte loss during MTZ treatment, consistent with studies in the mouse.

Targeting Mertk-signalling more specifically using the inhibitor UNC2025 did not exacerbate demyelination in this model, but did inhibit oligodendrocyte repopulation during the remyelinating phase, indicating that signalling via Mertk is limited to promotion of remyelination in this *Xenopus* model. Microglia containing myelin debris have previously been identified in this model [48], suggesting that pharmacological inhibition of Mertk with UNC2025 may impair remyelination by inhibiting clearance of debris in *Xenopus*. This is in line with previous work in rodents demonstrating inhibition of OPC differentiation by myelin debris [49], as well our present data in Mertk cKO murine cells. Confirmation of this hypothesis will require specific assessment of differences in clearance of myelin in the presence or absence of Mertk inhibition in this model. It will further be important to replicate this data either with genetic ablation of Mertk or with a more specific pharmacological inhibitor of Mertk, given UNC2025 is known to also inhibit Flt3.

This study has provided important insights into the role of microglial Mertk in myelination in both rodents and amphibians. Our experiments in Mertk cKO mice are the first to identify microglial Mertk as a mediator of developmental oligodendrogenesis and myelination. The induction of demyelination in the absence of microglial Mertk resulted in a worsened course of demyelination and altered the dynamics of recovery. Furthermore, the *Xenopus laevis* studies are the first to show conservation of the promyelinating effect of TAM signalling in this species. Using the *Tg*(*mbp:GFP-NTR)* tadpole model, we are now in a position to begin rapid screening of small molecule Mertk agonists for promyelinating effects, which will be a valuable tool for testing potential promyelinating drugs in the future. Together, these findings open new avenues of investigation for further research, not only in TAM receptor biology, but also the microglial interactions that underpin myelination in health and disease.

## Materials and methods

### Chemicals and reagents

All chemicals and reagents were purchased from Sigma-Aldrich (St. Louis, Missouri, USA) unless specified otherwise. All tissue culture flasks and plates were purchased from Corning (Corning, New York, USA) and Thermo Scientific (Waltham, Massachusetts, USA), respectively.

### Animal resources

The *Mertk-floxed* line was constructed in collaboration with Dr. Renate Lewis of the Transgenic Vectors Core at the Hope Center for Neurological Disorders, Department of Neurology, Washington University School of Medicine, St. Louis, MO, United States. Exon 2 of the *Mertk* gene was targeted for conditional deletion to introduce early stop codons, excluding the possibility for any dominant negative effects of a kinase dead Mertk receptor. A loxP site was introduced by homologous recombination into a non-conserved region within a mouse 129/SvEv bacterial artificial chromosome (BAC) containing the *Mertk* genomic locus, 5’ to exon 2. A *frt*-flanked pGK-neomycin expression cassette was introduced 3’ of exon 2 into a non-conserved region to provide for positive selection in ES cells. A second loxP site was also introduced to flank exon 2 with loxP sites. A ~ 10.6-kb fragment including the floxed region, and the *frt*-flanked pGK-neomycin cassette was generated from the modified BAC via gap repair into a vector containing a diphtheria toxin cassette. The diphtheria toxin cassette provides for negative selection in embryonic stem (ES) cells. The targeting vector was linearised for ES cell electroporation with Ascl. Electroporation and selection of ES cells, microinjection and generation of *Mertk*^*fl/fl*^ chimeric mice were performed at the Mouse Genetics Core, Washington University School of Medicine, St. Louis, MO, United States. *Mertk*^*fl/fl*^, *Mertk*^*fl/wt*^* and Mertk*^*wt/wt*^ were born at expected Mendelian frequencies. These mice all appeared healthy and viable, displaying no abnormal phenotype. Prior to undertaking experiments, Mertk-floxed mice were fully backcrossed to the C57Bl/6 background. Backcrossing was confirmed using microsatellite testing, with mice confirmed as homozygous C57Bl/6 at all non-linked markers.

Mertk was conditionally deleted from microglia by crossing Mertk^fl/fl^ animals with Cx3Cr1 cre knock-in line (C57BL/6-Cx3cr1 < tm1.1(cre)Jung > /Orl Cx3Cr1^cre^) obtained from the European Mouse Mutant Archive repository (Infrafrontier, France). All mouse lines were maintained on a C57Bl/6 background and housed in a specific pathogen free environment. Male and female mice were used in all experiments, although group sizes were not specifically powered to allow stratification of final data by sex.

*Xenopus laevis* [*Tg*(*mbp:GFP-NTR)*] animals were bred from lines previously established at the Institut de Cerveau (ICM, Paris Brain Institute), Paris, France. Staging of animals was undertaken according to the Nieuwkoop and Faber (NF) stages of *Xenopus laevis* development [50].

### Assessment of cell proliferation in vivo

To identify cells undergoing proliferation in mouse tissue, mice were administered the thymidine analogue EdU at the same time each day (50 mg/kg, E10415, Thermofisher, Waltham, Massachusetts, USA) via intraperitoneal (IP) injection, for three consecutive days prior to collection. Twenty-four hours following the final EdU injection, animals were killed for tissue collection. EdU was detected using Click-iT™ Plus EdU Alexa Fluor™ 594 Imaging Kit (C10639, Invitrogen), as per manufacturer’s instructions.

### Purification of microglia and oligodendrocyte progenitor cells

Microglia and oligodendrocyte progenitor cells (OPCs) were purified from young mice (P7) using sequential immunopanning as previously described [51, 52]. Microglia were disrupted in Buffer RLT Plus (Qiagen, Hilden, Germany) on the plate using a cell scraper for subsequent use in quantitative PCR experiments. OPCs were released from the antibody-coated plates via trypsinisation for assessment of proliferation.

### Assessment of OPC proliferation in vitro

Purified OPCs were cultured on PDL-coated glass coverslips in DMEM-SATO with PDGF and NT3 to promote proliferation [52]. To identify proliferating cells bromodeoxyuridine (BrdU, 10 µM) was added to the culture media for 48 h. At the end of 48 h, cells which had incorporated BrdU identified using immunostaining as previously described using an anti-BrdU antibody (1:250, ab6326, Abcam, Cambridge, UK) [20]. Coverslips were mounted onto glass microscope slides using Dako fluorescent mounting medium (Agilent Technologies, Santa Clara, California, USA).

### Quantitative real-time polymerase chain reaction (qPCR)

RNA was extracted from purified microglia using the Qiagen Micro RNeasy Kit (Qiagen, Hilden, Germany), as per manufacturer’s instructions. RNA and protein were extracted from fresh-frozen tissue using the PARIS Kit (AM1921, Invitrogen), and DNA was removed using the DNA-free™ Kit (AM1906, Invitrogen), as per manufacturer’s instructions. Reverse transcription PCR (RT-PCR) was performed using TaqMan™ Reverse Transcription Reagents (Applied Biosystems, Massachusetts, USA), as per manufacturer’s instructions. Real-time quantitative polymerase chain reaction (qPCR) was then performed using SYBR™ Green PCR Master Mix (Applied Biosystems, Cat# 4309155). Samples were run in duplicates, with reactions made up as per Table 1, run on a Viia 7 Real Time PCR System (Applied Biosystems). Relative fold change in expression was calculated using the ∆∆CT method, using 18S to normalise for RNA input.

**Table 1:** Primer sequences for qPCR

Target	Forward primer	Reverse primer
*18S*	CGGCTACCACATCCAAGGAA	GCTGGAATTACCGCGGCT
*Cnp*	TGCTGGACTGTACAACCAAAT TC	TCCTGGTGGGCGTATTCTTC
*Fgf2*	AGCGGCTCTACTGCAAGAAC	CGTGTGGGTCGCTCTTCT
*Gas6*	GCTGCAGCTTCGGTACAATG	ACATGCCGTGGTTGATGGTT
*Gpnmb*	CCATGCTTCATCTGCCTTCC	CACACACGCACCATACACAA
*Igf1*	AGACAGGCATTGTGGATGAG	TTCAGTGGGGCACAGTACAT
*IL1β*	GCCGTCTTTCATTACACAGG	GAGAACCAAGCAACGACAAA
*Lgals3*	TCAGCCTTCCCCTTTGAGAG	GCAGTAGGTGAGCATCGTTG
*Lif*	CCAACAACGTGGAAAAGCTA	CCATGGAAAGATGGGAAGTC
*Mbp*	CCCGTGGAGCCGTGATC	TCTTCAAACGAAAAGGGACGA
*Pdgfra*	ACCTCACCTGGACCTCTTTC	TGGCCAAAGTGGAGTATGTC
*Pros1*	TTTGATGATAGTGCATTGCAAGTG	CGTCTGATGCACCCCTGAA
*Spp1*	TCACATGAAGAGCGGTGAGT	CCCTTTCCGTTGTTGTCCTG

### Tissue collection from neonate and adult mice

Mice were anaesthetised using sodium pentobarbitone (80–100 mg/kg in MT-PBS, Troy Ilium Laboratories, New South Wales, Australia) administered via IP injection. Animals were immediately decapitated if no perfusion was required. If perfusion and fixation were required, animals were intracardially perfused with warm, sterile PBS, followed by cold, sterile, homemade 4% (w/v) PFA) or 4% (w/v) EM grade PFA (Electron Microscopy Solutions, Pennsylvania, USA) in PBS. Brain tissue was removed using forceps and fine scissors.

### Transmission electron microscopy (TEM)

For TEM, brain tissue was removed sliced in a 1-mm coronal rodent brain matrix. Tissue was incubated in Karnovsky’s solution at 4 °C overnight. The corpus callosum was trimmed at the midline and washed with 0.1 M sodium cacodylate buffer (pH 7.4). Samples were post-fixed in 1.5% potassium ferrocyanide and 1% osmium tetroxide in 0.1 M sodium cacodylate buffer, followed by dehydration in solutions of 50, 70, 90, 95 and 100% ethanol, and then 100% acetone and 1:1 Spurr’s resin/100% acetone. Tissue was incubated in 100% Spurr’s resin and then embedded in resin overnight at 70 °C to polymerise. Semi-thin sections (0.5 µm) were cut to confirm tissue orientation. Regions of interest were chosen, after which ultra-thin sections (90 nm) were cut using a diamond knife (Diatome, Austria) and UC7 ultramicrotome (Leica, Mannheim, Germany). Contrasting was performed with 0.15% uranyl acetate and lead citrate.

**Fig. 10:**
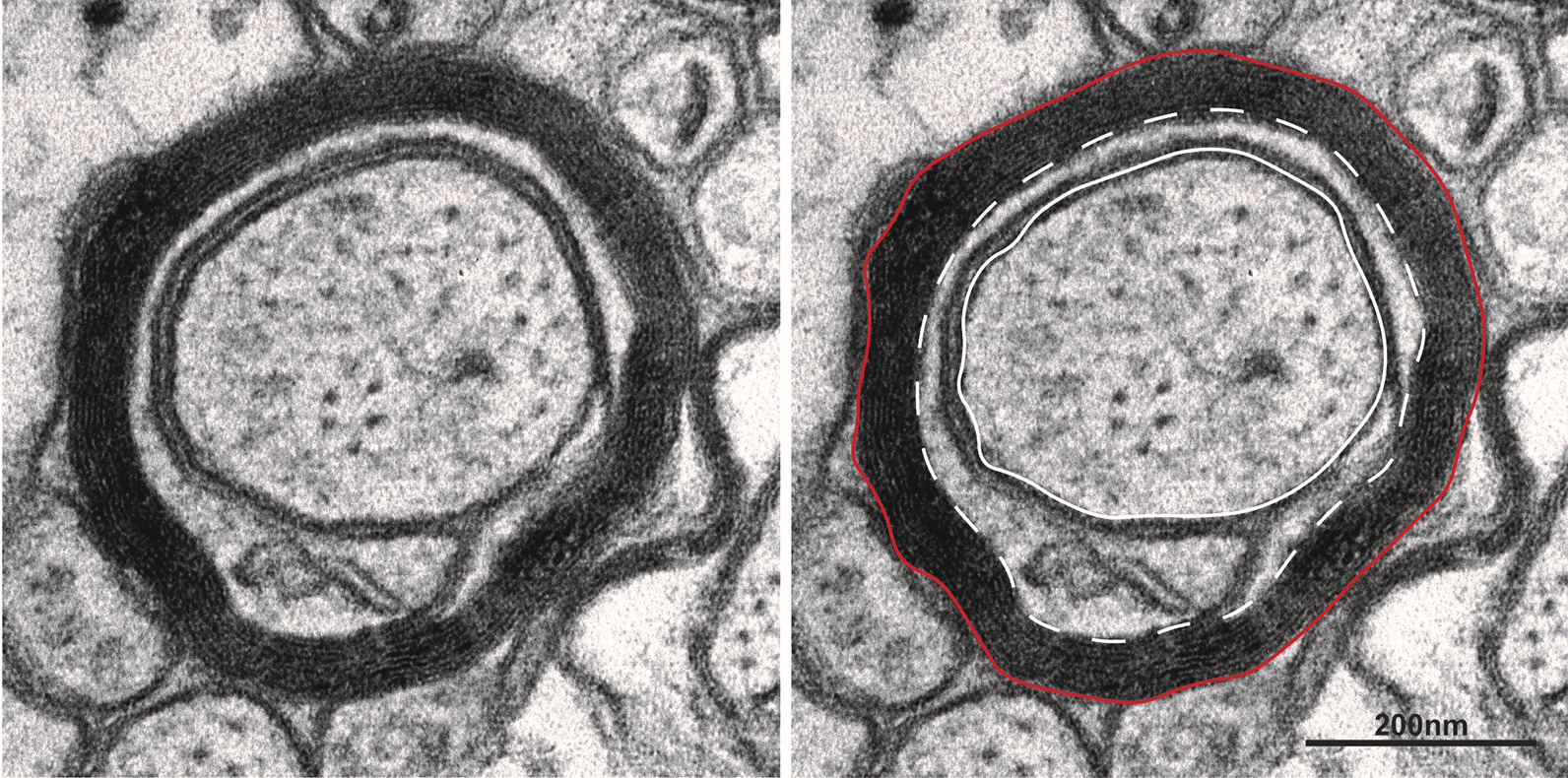
Myelinated axon measurements. Left: close-up image view of myelinated axon, unannotated. Right: same fibre with measured axon (solid white line), inner wrap (dotted white line) and outer wrap (solid red line). Scale bar represents 200 nm

For quantification of myelin gaps, high power images were captured on a JEM-1011 TEM (JEOL Ltd) at 50,000–200,000× magnification. Between 21 and 50 myelinated axons were counted per animal. For quantification of myelin thickness and myelinated axons, montage (3 × 3) images were taken on a JEM-1400 Flash TEM (JEOL Ltd, Tokyo, Japan) at 6000× magnification. Three to four images were analysed per animal. Measurements were performed using FIJI (Version 2.0.0) [53]. For each myelinated axon in a region of interest, area measurements were taken for the axon, and inner- and outermost myelin wraps (Fig. 10). Measured area values were used to determine the diameter of an equivalent circle, from which myelin thickness was calculated. Myelin thickness was expressed as a direct measurement as measures such as g-ratio include inner tongue which has previously been shown to vary with loss of microglia [3].

### Induction of demyelination in mice

Demyelination was induced in 8- to 10-week-old mice using the toxin cuprizone (bis-cyclohexanone-oxaldihydrazone), provided at 0.2% (w/w) in the form of pre-made pellets (Envigo, Huntingdon, UK) or mixed into powdered high-fat feed (catalogue no. 10212, Barastoc/Ridley, Melbourne, Victoria, Australia). Mice were housed in groups of 2 and fed 0.2% cuprizone feed ad libitum for up to 5 weeks. Body weight was monitored twice a week and animals were culled if weight loss exceeded 20% of original weight.

### Tissue preparation for immunohistochemistry

Following removal, adult mouse brain tissue was post-fixed for up to 4 h in 4% PFA in MT-PBS, on ice, and then transferred to 30% sucrose (w/v) in PBS for dehydration at 4 °C. Tissue was embedded in Optimal Cutting Temperature (OCT, Sciegen, Singapore) in an isopentane (RCI Labscan Limited, Bangkok, Thailand) bath on dry ice, then stored at − 80 °C. Coronal cryosections (10 µm) were cut using Leica CM3050 S cryostat (Leica, Victoria, Australia) and mounted onto SuperFrost Plus microscope slides (Menzel Gläser, Braunschweig, Germany). Slides were then stored at − 80 °C.

### Fluorescent immunohistochemistry

Tissue was rehydrated in MT-PBS prior to incubation in blocking buffer (Table 2) for 1 h at room temperature. Tissue was then incubated with primary antibodies in blocking buffer overnight at room temperature. Slides were then washed 3 × in mouse tonicity (MT)-PBS with gentle shaking and then incubated with relevant secondary antibodies and Hoechst 33342 nuclear dye (1:2000, H3570, Invitrogen) (Table 3) in blocking buffer for 1 h at room temperature. Slides were washed 3 × in MT-PBS and then coverslips were mounted using Dako Fluorescent Mounting Medium (Agilent Technologies, Santa Clara, California, USA). Slides were stored at 4 °C.

**Table 2:** Primary antibodies and blocking buffers for immunohistochemistry

Target	Host species	Dilution	Manufacturer, catalogue #	Blocking buffer
Mertk	Rat	1:50	eBioscience™, DS5MMER	0.1% X-100 Triton/10% normal serum in MT-PBS
Iba1	Rabbit	1:1000	Wako, 019-19741	0.3% X-100 Triton/10% normal serum in MT-PBS
PDGFRα	Goat	1:50	R&D Systems, AF1062	
CC1	Mouse	1:100	Merck Millipore, OP80	
dMBP	Rabbit	1:500	Sigma-Aldrich, AB5864

**Table 3:** Secondary antibodies for immunohistochemistry

Target species	Host species	Dilution	Conjugated fluorophore	Manufacturer, catalogue #
Rabbit	Goat	1:200	AlexaFluor 488	Jackson ImmunoResearch, West Grove, Pennsylvania, USA, 111-545-003
Rat	Goat	1:200	AlexaFluor 594	Invitrogen, A11007
Mouse	Goat	1:200	AlexaFluor 594	Jackson ImmunoResearch, 115-585-207
Rabbit	Goat	1:200	FITC	Jackson ImmunoResearch, 111-095,003
Goat	Donkey	1:200	TRITC	Jackson ImmunoResearch, 705-025-003
Rabbit	Donkey	1:200	AlexaFluor 488	Invitrogen, A21206

Immunofluorescence images were collected using either a Zeiss Axioplan 2 (Zeiss, Oberkochen, Germany) or Zeiss Axio Imager M2 fluorescent microscope with apotome. Cell counts were performed using Adobe Photoshop CC 2018–2022 (San Jose, California, USA), using the Count tool.

### Generation of mixed glial cultures

Neonate mice (P0-2) were anaesthetised by isoflurane inhalation and decapitated. Mixed glial cultures were generated as described by McCarthy and de Vellis [54], with some modifications. Mixed glial cultures were incubated at 37 °C/5% CO_2_ for 10–14 days. Culture media was replaced on days 1 and 4 after initial seeding.

### Preparation of crude human myelin debris for phagocytosis assay

Crude human myelin was obtained from Percoll gradient interface as described by [55]. Myelin was then purified and fluorescently labelled as described previously [56]. Myelin debris was incubated with 2 mM pHrodo iFL Green STP Ester (amine-reactive) (P36012, Invitrogen) or 50 µM CellTrace CFSE dye (C34554, Invitrogen) for 30–60 min at room temperature, washed, and then resuspended to 100 mg/ml with sterile PBS.

### Phagocytosis assay

Phagocytosis assays were performed in mixed glial cultures. Labelled human myelin debris was added to cultures (final conc. 1 mg/ml), with and without rhGAS6 treatment (final conc. 100 ng/ml, R&D Systems) and incubated at 37 °C/5% CO_2_ for 1 h to allow for engulfment to occur. After incubation, excess media was aspirated, and flasks were washed with media to remove excess non-engulfed myelin debris. Microglia were harvested from mixed glial cultures via mechanical dissociation, and resuspended into smaller volume for flow cytometry.

### Flow cytometry

Cells were stained for 30 min at 4 °C in anti-Cx3Cr1-PE/anti-Mertk-APC and anti-Cx3Cr1-PE/anti-REA-APC antibody cocktails (Table 4), respectively, covered from light. Following incubation, cells were washed in FACS wash buffer and resuspended in wash buffer. Samples were analysed on a Cytoflex S flow cytometer (Beckman Coulter).

**Table 4:** Antibodies used for flow cytometry

Target	Conjugated fluorophore	Antibody	Dilution
Mouse Cx3Cr1	Phycoerythrin (PE)	BioLegend, cat no: 149006	1:200
Mouse Mertk	Allophycocyanin (APC)	Miltenyi Biotec, order no.: 130-107-479	1:10
Recombinant human IgG1 (isotype control)	APC	Miltenyi Biotec, order no.: 130-104-630	1:10

### Induction of demyelination in *Xenopus laevis* tadpoles

Demyelination was induced in stage 50–55 *Tg*(*mbp:GFP-NTR)* tadpoles by the addition of the prodrug metronidazole (MTZ, 10 mM in 0.05% DMSO) to aquaria water (0.1X MMR). MTZ-treated water was changed every 72 h during the treatment course. After 10 days, MTZ-treated tadpoles were moved to standard, filtered, dechlorinated water to recover for 3 days. Untreated control animals were housed in standard water for the entirety of the experiment. Animals were housed in groups of up to 10 animals per 600 ml in oxygenated aquariums, covered from light, as MTZ is light-sensitive.

### Quantification of GFP-positive cells in *Xenopus laevis* optic nerve

GFP^+^ cells in *Tg*(*mbp:GFP-NTR)* tadpoles were live-imaged using a fluorescence macroscope (Nikon Multizoom AZ100M). Tadpoles were anaesthetised in 0.01% MS222 solution until the animals were unconscious and no tail reflex was observed. Animals were transferred to a small drop of 0.01% MS222 on a clear plastic surface, and then GFP^+ve^ oligodendrocytes were counted along the optic nerve after the chiasma to the base of the retina.

### Intracerebroventricular (ICV) delivery of rhGAS6 into Xenopus laevis tadpoles

Intracerebroventricular (ICV) injections into tadpoles were performed using a modified glass capillary tube and Nanoject III programmable nanoliter injector (Drummond Scientific, Pennsylvania, USA). Tadpoles were placed onto a damp paper towel and a small hole was punctured into the head using a modified Pasteur pipette and a small piece of wire. Tadpoles received a 25 nl volume of rhGAS6 (1 µg/ml, R&D Systems) in 3% D-(+)-glucose solution, or saline control, injected at a rate of 100 nl/s. Injection solutions were mixed with a small amount of Fast Green FCF dye to visualise injection into the ventricle. After injection, animals were returned to aquaria.

### UNC2025 treatment

Tadpoles were treated with 0.5 µM UNC2025 (Item no. 16613, Cayman Chemicals, Ann Arbor, Michigan, USA) in aquaria water, changed every 24–72 h.

### Statistical analyses

All statistical analysis was performed using GraphPad Prism 9 (GraphPad Software, San Diego, California, USA). For any comparisons at a single-time point, unpaired *t*-tests were performed, except in the case of uneven variances, where Welch’s *t*-test was performed. Categorical data (e.g. myelin gap data) were analysed using Fisher's exact test. Myelin thickness data were analysed by simple linear regression. The relationship between axon calibre and myelin thickness was assessed using simple linear regression. Multi-factorial experiments were analysed using 2-way ANOVA, followed by pre-planned multiple comparisons if main effects were observed. As only pre-planned multiple comparisons were performed results from Fisher's least significant difference approach are reported for planned tests. The test statistic and confidence interval of all planned tests are reported in Additional file 6: Table S1.

### Supplementary Information

**Additional file 1: Figure S1.** The densities of microglia and oligodendrocyte lineage cells in adult mice are not altered by the loss of microglial Mertk. **A** Representative immunofluorescence images of corpus callosum tissue from Mertk WT and cKO animals. Scale bar represents 50 µm. Densities of Iba1^+^ microglia (**B**), Olig2^+^/PDGFRα^+^ OPCs (**C**) and CC1^+^ oligodendrocytes (**F**) were not different between genotypes (Student’s *t*-test; *P* > 0.05). *n = *3–4 biological replicates per genotype. Data represent mean ± SD.

**Additional file 2: Figure S2.** OPCs do not express Mertk. OPCs were purified from P7 Mertk WT and cKO neonate brains using immunopanning. Cx3Cr1 and Mertk expression was assessed using flow cytometry. Cells were gated based on cell bodies and single cells (not shown), and then for Cx3Cr1 and Mertk. **A** Cx3Cr1 was detected in a small subset of OPCs (4–8%), as compared to unstained control cells. **B** Of Cx3Cr1^+^ cells, almost no Mertk^+ve^ cells were observed in each genotype. **C** There was a significant decrease in the percentage of Cx3Cr1^+ve^ cells in Mertk cKO OPCs, due to haploinsufficiency from Cre recombinase knock-in. There were few Mertk^+ve^ cells as a percentage of (**D**) Cx3Cr1^+ve^ cells and (**E**) total cells (*P* > 0.05). *n = *2–3 biological replicates. Data represent mean ± SD. Statistical significance determined using unpaired *t*-tests.

**Additional file 3: Figure S3.** Proliferation is not altered in cultures of OPCs purified from Mertk cKO mice. **A** Representative immunofluorescence images of purified OPCs showing BrdU^+ve^ (red) nuclei. Nuclei counterstained with Hoechst (blue). Scale bar represents 50 µm. The proportion of proliferating cells is quantified in **B**. The proportion of BrdU^+ve^ cells is similar between OPCs derived from Mertk WT or cKO mice. *n = *3–5 biological replicates from two experiments. Data represent mean ± SD. Statistical significance was determined using an unpaired *t*-test. **C** Microglial expression of candidate genes were similar between purified microglia derived from Mertk WT and cKO mice (*P* > 0.05). Relative gene expression presented on log_2_ scale. *n = *3–5 biological replicates. Data represent mean ± SD. Statistical significance was determined using unpaired Student's *t*-tests.

**Additional file 4: Figure S4.** Microglial deficiency of Mertk does not alter cellular dynamics during remyelination. **A** Representative immunofluorescence images of the corpus callosum tissue of Mertk WT and cKO mice following 2 and 4 weeks recovery on standard chow. Hoechst-labelled nuclei in cyan. Scale bar represents 50 µm. The density of Iba1^+ve^ microglia (**B**), PDGFRα^+ve^ OPCs (**C**) and CC1^+ve^ oligodendrocytes was not altered in Mertk cKO compared with Mertk WT mice. *n = *4 biological replicates. Data represent means ± SD. Statistical significance was determined using two-way ANOVA with Fisher's LSD.

**Additional file 5: Figure S5.** *Ifn*γ gene expression is not altered by loss of microglial Mertk during cuprizone-mediated demyelination or subsequent remyelination. **A** Demyelination was induced in Mertk WT and cKO using cuprizone (0.2% w/w). Mice were collected at demyelination time-points: 3wk CPZ, 4wk CPZ and 5wk CPZ. After cuprizone-challenge, cohorts of mice were returned to standard chow for tissue collection at remyelination time-points: 4wk CPZ + 2wk rec and 5wk CPZ + 2wk rec. Corpus callosum tissue was dissected and processed for RNA extraction and qPCR. **B** Dissected corpus callosum tissue from cKO and WT animals displayed no differences in *Ifng* gene expression gene expression at any time-point (*P* > 0.05). Relative gene expression presented on log2 scale. *n = *3–5 biological replicates. All data represent mean ± SD. Data from 3 to 5 weeks CPZ time-points analysed by unpaired *t*-tests. Data from 2–3 weeks recovery time-points analysed by Welch’s *t* test.

**Additional file 6. Table S1.** Test statistic and confidence intervals of statistical analyses.

## Data Availability

All data generated or analysed during this study are included in this published article and its supplementary information files.

## References

[1] Hagemeyer N, Hanft K-M, Akriditou M-A, Unger N, Park ES, Stanley ER (2017). Microglia contribute to normal myelinogenesis and to oligodendrocyte progenitor maintenance during adulthood. Acta Neuropathol.

[2] Wlodarczyk A, Holtman IR, Krueger M, Yogev N, Bruttger J, Khorooshi R (2017). A novel microglial subset plays a key role in myelinogenesis in developing brain. EMBO J.

[3] McNamara NB, Munro DAD, Bestard-Cuche N, Uyeda A, Bogie JFJ, Hoffmann A (2023). Microglia regulate central nervous system myelin growth and integrity. Nature.

[4] Paolicelli RC, Bolasco G, Pagani F, Maggi L, Scianni M, Panzanelli P (2011). Synaptic pruning by microglia is necessary for normal brain development. Science.

[5] Weinhard L, di Bartolomei G, Bolasco G, Machado P, Schieber NL, Neniskyte U (2018). Microglia remodel synapses by presynaptic trogocytosis and spine head filopodia induction. Nat Commun.

[6] Marín-Teva JL, Dusart I, Colin C, Gervais A, van Rooijen N, Mallat M (2004). Microglia promote the death of developing Purkinje cells. Neuron.

[7] Cunha MI, Su M, Cantuti-Castelvetri L, Müller SA, Schifferer M, Djannatian M (2020). Pro-inflammatory activation following demyelination is required for myelin clearance and oligodendrogenesis. J Exp Med.

[8] Hughes AN, Appel B (2020). Microglia phagocytose myelin sheaths to modify developmental myelination. Nat Neurosci.

[9] Djannatian M, Radha S, Weikert U, Safaiyan S, Wrede C, Deichsel C (2023). Myelination generates aberrant ultrastructure that is resolved by microglia. J Cell Biol.

[10] Binder MD, Kilpatrick TJ (2009). TAM receptor signalling and demyelination. Neuro Signals.

[11] Scott RS, McMahon EJ, Pop SM, Reap EA, Caricchio R, Cohen PL (2001). Phagocytosis and clearance of apoptotic cells is mediated by MER. Nature.

[12] Healy LM, Perron G, Won S-Y, Michell-Robinson MA, Rezk A, Ludwin SK (2016). MerTK is a functional regulator of myelin phagocytosis by human myeloid cells. J Immunol.

[13] Fourgeaud L, Través PG, Tufail Y, Leal-Bailey H, Lew ED, Burrola PG (2016). TAM receptors regulate multiple features of microglial physiology. Nature.

[14] Zizzo G, Hilliard BA, Monestier M, Cohen PL (2012). Efficient clearance of early apoptotic cells by human macrophages requires M2c polarization and MerTK induction. J Immunol.

[15] Camenisch TDT, Koller BHB, Earp HSH, Matsushima GKG (1999). A novel receptor tyrosine kinase, Mer, inhibits TNF-alpha production and lipopolysaccharide-induced endotoxic shock. J Immunol (Baltimore, Md : 1950).

[16] Ma GZM, Stankovich J, Kilpatrick TJ, Binder MD, Field J, The Australia and New Zealand Multiple Sclerosis Genetics Consortium (ANZgene) (2010). Polymorphisms in the receptor tyrosine kinase MERTK gene are associated with multiple sclerosis susceptibility. PLoS ONE.

[17] Binder MD, Fox AD, Merlo D, Johnson LJ, Giuffrida L, Calvert SE (2016). Common and low frequency variants in MERTK are independently associated with multiple sclerosis susceptibility with discordant association dependent upon HLA-DRB1*15:01 status. PLoS Genet.

[18] Grommes C, Lee CYD, Wilkinson BL, Jiang Q, Koenigsknecht-Talboo JL, Varnum B (2008). Regulation of microglial phagocytosis and inflammatory gene expression by Gas6 acting on the Axl/Mer family of tyrosine kinases. J Neuroimmune Pharmacol.

[19] Ji R, Tian S, Lu HJ, Lu Q, Zheng Y, Wang X (2013). TAM receptors affect adult brain neurogenesis by negative regulation of microglial cell activation. J Immunol.

[20] Binder MD, Cate HS, Prieto AL, Kemper D, Butzkueven H, Gresle MM (2008). Gas6 deficiency increases oligodendrocyte loss and microglial activation in response to cuprizone-induced demyelination. J Neurosci.

[21] Hoehn HJ, Kress Y, Sohn A, Brosnan CF, Bourdon S, Shafit-Zagardo B (2008). Axl^-/-^ mice have delayed recovery and prolonged axonal damage following cuprizone toxicity. Brain Res.

[22] Ray AK, DuBois JC, Gruber RC, Guzik HM, Gulinello ME, Perumal G (2017). Loss of Gas6 and Axl signaling results in extensive axonal damage, motor deficits, prolonged neuroinflammation, and less remyelination following cuprizone exposure. Glia.

[23] Blades F, Aprico A, Akkermann R, Ellis S, Binder MD, Kilpatrick TJ (2018). The TAM receptor TYRO3 is a critical regulator of myelin thickness in the central nervous system. Glia.

[24] Shen K, Reichelt M, Kyauk RV, Ngu H, Shen Y-AA, Foreman O (2021). Multiple sclerosis risk gene Mertk is required for microglial activation and subsequent remyelination. Cell Rep.

[25] Yona S, Kim K-W, Wolf Y, Mildner A, Varol D, Breker M (2013). Fate mapping reveals origins and dynamics of monocytes and tissue macrophages under homeostasis. Immunity.

[26] Snaidero N, Velte C, Myllykoski M, Raasakka A, Ignatev A, Werner HB (2017). Antagonistic functions of MBP and CNP establish cytosolic channels in CNS myelin. Cell Rep.

[27] Watson AES, de Almeida MMA, Dittmann NL, Li Y, Torabi P, Footz T (2021). Fractalkine signaling regulates oligodendroglial cell genesis from SVZ precursor cells. Stem Cell Rep.

[28] Li Q, Cheng Z, Zhou L, Darmanis S, Neff NF, Okamoto J (2019). Developmental heterogeneity of microglia and brain myeloid cells revealed by deep single-cell RNA sequencing. Neuron.

[29] Kaya F, Mannioui A, Chesneau A, Sekizar S, Maillard E, Ballagny C (2012). Live imaging of targeted cell ablation in Xenopus: a new model to study demyelination and repair. J Neurosci.

[30] Sherafat A, Pfeiffer F, Reiss AM, Wood WM, Nishiyama A (2021). Microglial neuropilin-1 promotes oligodendrocyte expansion during development and remyelination by trans-activating platelet-derived growth factor receptor. Nat Commun.

[31] Rosenbluth J, Nave K-A, Mierzwa A, Schiff R (2006). Subtle myelin defects in PLP-null mice. Glia.

[32] Weil M-T, Möbius W, Winkler A, Ruhwedel T, Wrzos C, Romanelli E (2016). Loss of myelin basic protein function triggers myelin breakdown in models of demyelinating diseases. Cell Rep.

[33] Peters A (2002). The effects of normal aging on myelin and nerve fibers: a review. J Neurocytol.

[34] Payne SC, Bartlett CA, Harvey AR, Dunlop SA, Fitzgerald M (2012). Myelin sheath decompaction, axon swelling, and functional loss during chronic secondary degeneration in rat optic nerve. Invest Ophthalmol Vis Sci.

[35] Rosenbluth J (1980). Central myelin in the mouse mutant shiverer. J Comp Neurol.

[36] Boison D, Bussow H, D’Urso D, Muller H, Stoffel W (1995). Adhesive properties of proteolipid protein are responsible for the compaction of CNS myelin sheaths. J Neurosci.

[37] Liu A, Li J, Marin-Husstege M, Kageyama R, Fan Y, Gelinas C (2006). A molecular insight of Hes5-dependent inhibition of myelin gene expression: old partners and new players. EMBO J.

[38] Marin-Husstege M, He Y, Li J, Kondo T, Sablitzky F, Casaccia-Bonnefil P (2006). Multiple roles of Id4 in developmental myelination: predicted outcomes and unexpected findings. Glia.

[39] Ji R, Meng L, Jiang X, Cvm NK, Ding J, Li Q (2014). TAM receptors support neural stem cell survival, proliferation and neuronal differentiation. PLoS ONE.

[40] Butti E, Bacigaluppi M, Chaabane L, Ruffini F, Brambilla E, Berera G (2018). Neural stem cells of the subventricular zone contribute to neuroprotection of the corpus callosum after cuprizone-induced demyelination. J Neurosci.

[41] Xing YL, Röth PT, Stratton JAS, Chuang BHA, Danne J, Ellis SL (2014). Adult neural precursor cells from the subventricular zone contribute significantly to oligodendrocyte regeneration and remyelination. J Neurosci.

[42] Remaud S, Ortiz FC, Perret-Jeanneret M, Aigrot M-S, Gothié J-D, Fekete C (2017). Transient hypothyroidism favors oligodendrocyte generation providing functional remyelination in the adult mouse brain. Elife.

[43] Brousse B, Mercier O, Magalon K, Daian F, Durbec P, Cayre M (2021). Endogenous neural stem cells modulate microglia and protect against demyelination. Stem Cell Rep.

[44] Miner JJ, Daniels BP, Shrestha B, Proenca-Modena JL, Lew ED, Lazear HM (2015). The TAM receptor Mertk protects against neuroinvasive viral infection by maintaining blood-brain barrier integrity. Nat Med.

[45] Paredes I, Vieira JR, Shah B, Ramunno CF, Dyckow J, Adler H (2021). Oligodendrocyte precursor cell specification is regulated by bidirectional neural progenitor—endothelial cell crosstalk. Nat Neurosci.

[46] Tsiperson V, Li X, Schwartz GJ, Raine CS, Shafit-Zagardo B (2010). GAS6 enhances repair following cuprizone-induced demyelination. PLoS ONE.

[47] Gruber RC, Ray AK, Johndrow CT, Guzik H, Burek D, de Frutos PG (2014). Targeted GAS6 delivery to the CNS protects axons from damage during experimental autoimmune encephalomyelitis. J Neurosci.

[48] Sekizar S, Mannioui A, Azoyan L, Colin C, Thomas J-L, Pasquier DD (2015). Remyelination by resident oligodendrocyte precursor cells in a *Xenopus*
*laevis* inducible model of demyelination. Dev Neurosci-basel.

[49] Kotter MR, Li W-W, Zhao C, Franklin RJM (2006). Myelin impairs CNS remyelination by inhibiting oligodendrocyte precursor cell differentiation. J Neurosci.

[50] Faber J, Nieuwkoop PD, Nieuwkoop PD, Faber J (1994). Normal table of *Xenopus**laevis* (Daudin). A systematical and chronological survey of the development from the fertilized egg till the end of metamorphosis.

[51] Zhang Y, Sloan SA, Clarke LE, Caneda C, Plaza CA, Blumenthal PD (2016). Purification and characterization of progenitor and mature human astrocytes reveals transcriptional and functional differences with mouse. Neuron.

[52] Emery B, Dugas JC (2013). Purification of oligodendrocyte lineage cells from mouse cortices by immunopanning. Cold Spring Harb Protoc.

[53] Schindelin J, Arganda-Carreras I, Frise E, Kaynig V, Longair M, Pietzsch T (2012). Fiji: an open-source platform for biological-image analysis. Nat Methods.

[54] McCarthy KD, de Vellis J (1980). Preparation of separate astroglial and oligodendroglial cell cultures from rat cerebral tissue. J Cell Biol.

[55] Gosselin D, Skola D, Coufal NG, Holtman IR, Schlachetzki JCM, Sajti E (2017). An environment-dependent transcriptional network specifies human microglia identity. Science.

[56] Rolfe AJ, Bosco DB, Broussard EN, Ren Y (2017). In Vitro Phagocytosis of Myelin Debris by Bone Marrow-Derived Macrophages. J Vis Exp Jove.

