# Supplementary figures and images for: Mertk-expressing microglia influence oligodendrogenesis and myelin modelling in the CNS
Source: J Neuroinflammation. 2023 Nov 6;20:253. doi: 10.1186/s12974-023-02921-8 (PMC10626688; doi:10.1186/s12974-023-02921-8)

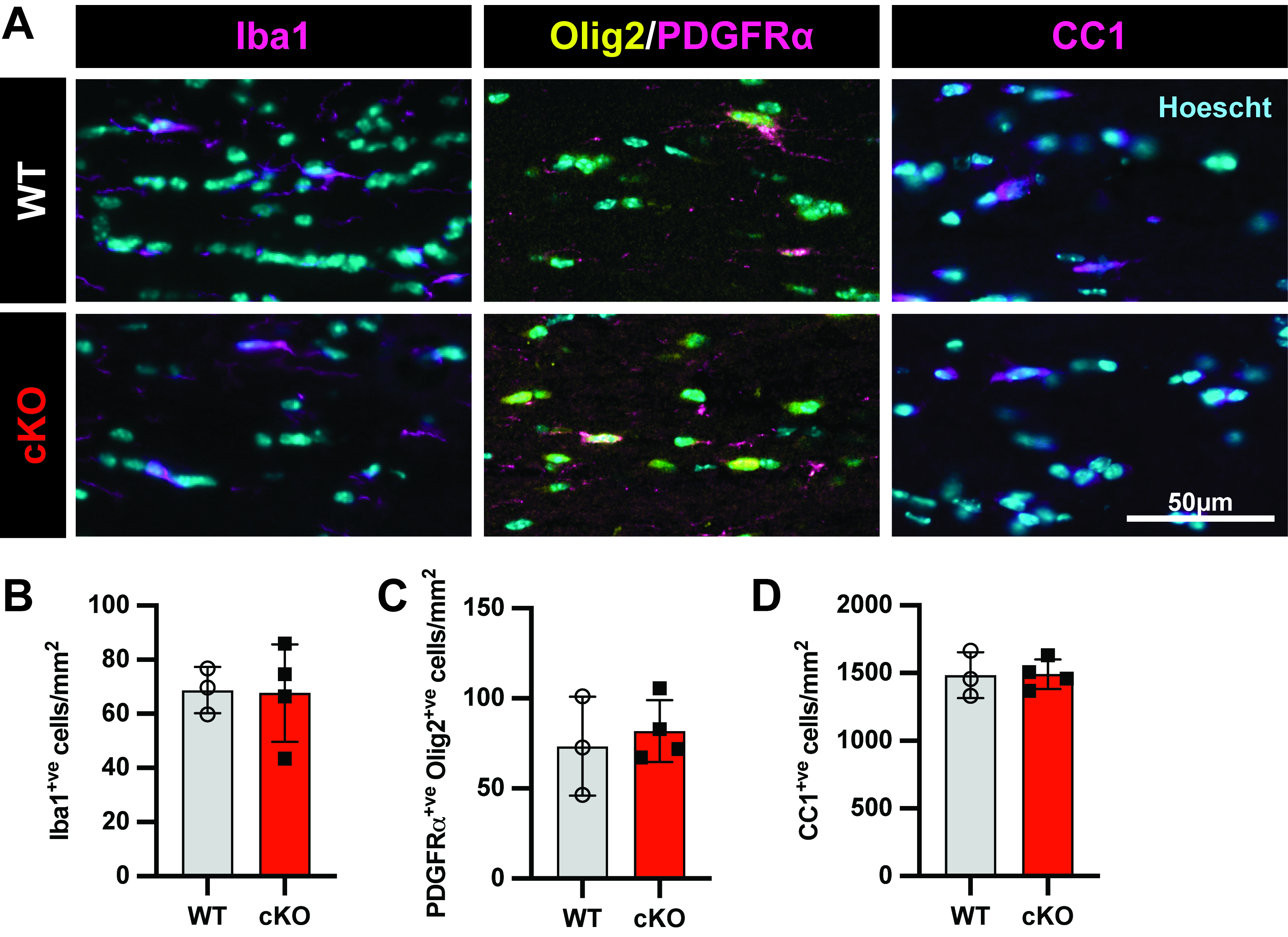

Supplement: Supplementary file 1 — Additional file 1: Figure S1. The densities of microglia and oligodendrocyte lineage cells in adult mice are not altered by the loss of microglial Mertk. A Representative immunofluorescence images of corpus callosum tissue from Mertk WT and cKO animals. Scale bar represents 50 µm. Densities of Iba1+ microglia (B), Olig2+/PDGFRα+ OPCs (C) and CC1+ oligodendrocytes (F) were not different between genotypes (Student’s t-test; P > 0.05). n = 3–4 biological replicates per genotype. Data represent mean ± SD. [file 12974_2023_2921_MOESM1_ESM.tif]

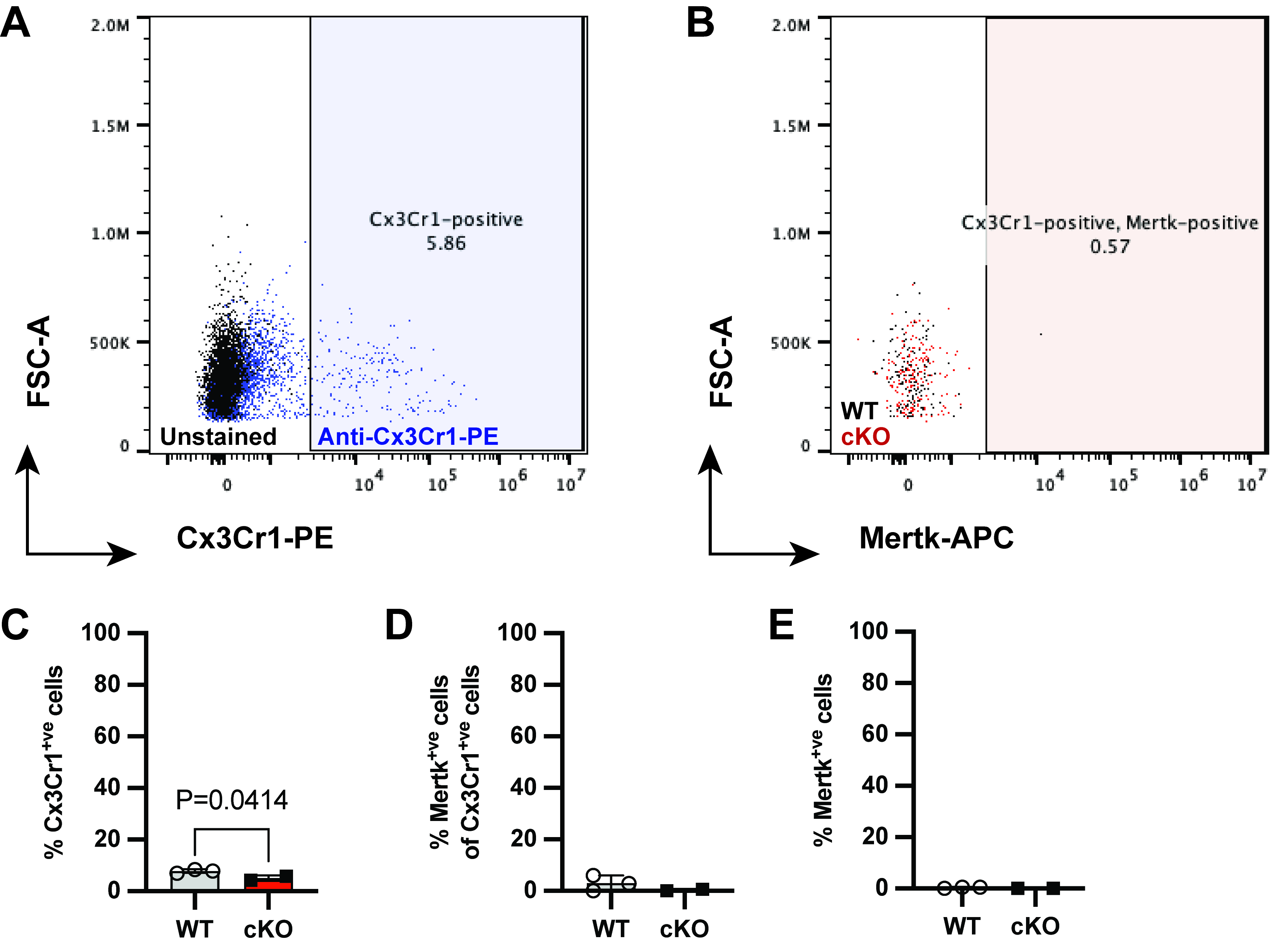

Supplement: Supplementary file 2 — Additional file 2: Figure S2. OPCs do not express Mertk. OPCs were purified from P7 Mertk WT and cKO neonate brains using immunopanning. Cx3Cr1 and Mertk expression was assessed using flow cytometry. Cells were gated based on cell bodies and single cells (not shown), and then for Cx3Cr1 and Mertk. A Cx3Cr1 was detected in a small subset of OPCs (4–8%), as compared to unstained control cells. B Of Cx3Cr1+ cells, almost no Mertk+ve cells were observed in each genotype. C There was a significant decrease in the percentage of Cx3Cr1+ve cells in Mertk cKO OPCs, due to haploinsufficiency from Cre recombinase knock-in. There were few Mertk+ve cells as a percentage of (D) Cx3Cr1+ve cells and (E) total cells (P > 0.05). n = 2–3 biological replicates. Data represent mean ± SD. Statistical significance determined using unpaired t-tests. [file 12974_2023_2921_MOESM2_ESM.tif]

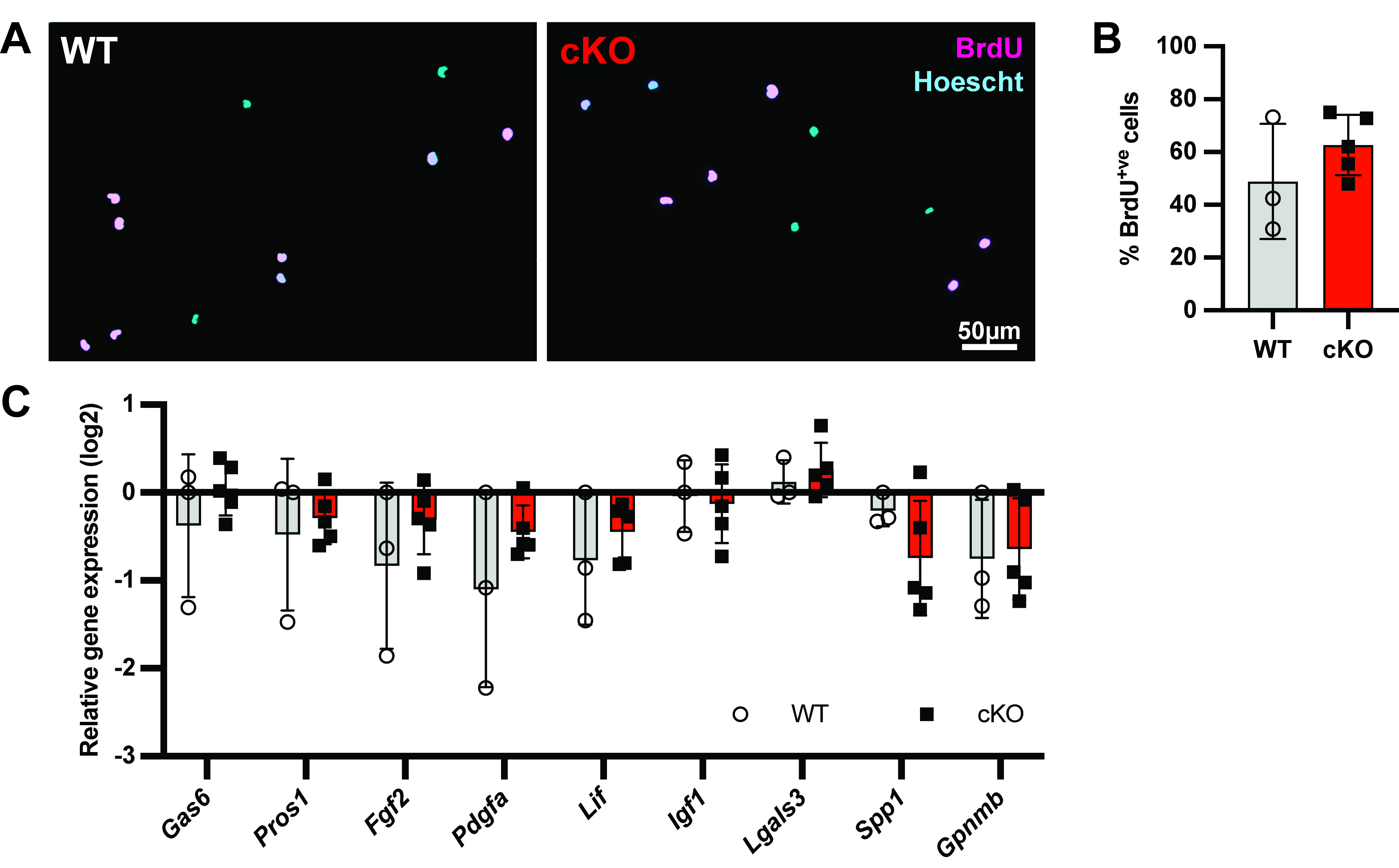

Supplement: Supplementary file 3 — Additional file 3: Figure S3. Proliferation is not altered in cultures of OPCs purified from Mertk cKO mice. A Representative immunofluorescence images of purified OPCs showing BrdU+ve (red) nuclei. Nuclei counterstained with Hoechst (blue). Scale bar represents 50 µm. The proportion of proliferating cells is quantified in B. The proportion of BrdU+ve cells is similar between OPCs derived from Mertk WT or cKO mice. n = 3–5 biological replicates from two experiments. Data represent mean ± SD. Statistical significance was determined using an unpaired t-test. C Microglial expression of candidate genes were similar between purified microglia derived from Mertk WT and cKO mice (P > 0.05). Relative gene expression presented on log2 scale. n = 3–5 biological replicates. Data represent mean ± SD. Statistical significance was determined using unpaired Student's t-tests. [file 12974_2023_2921_MOESM3_ESM.tif]

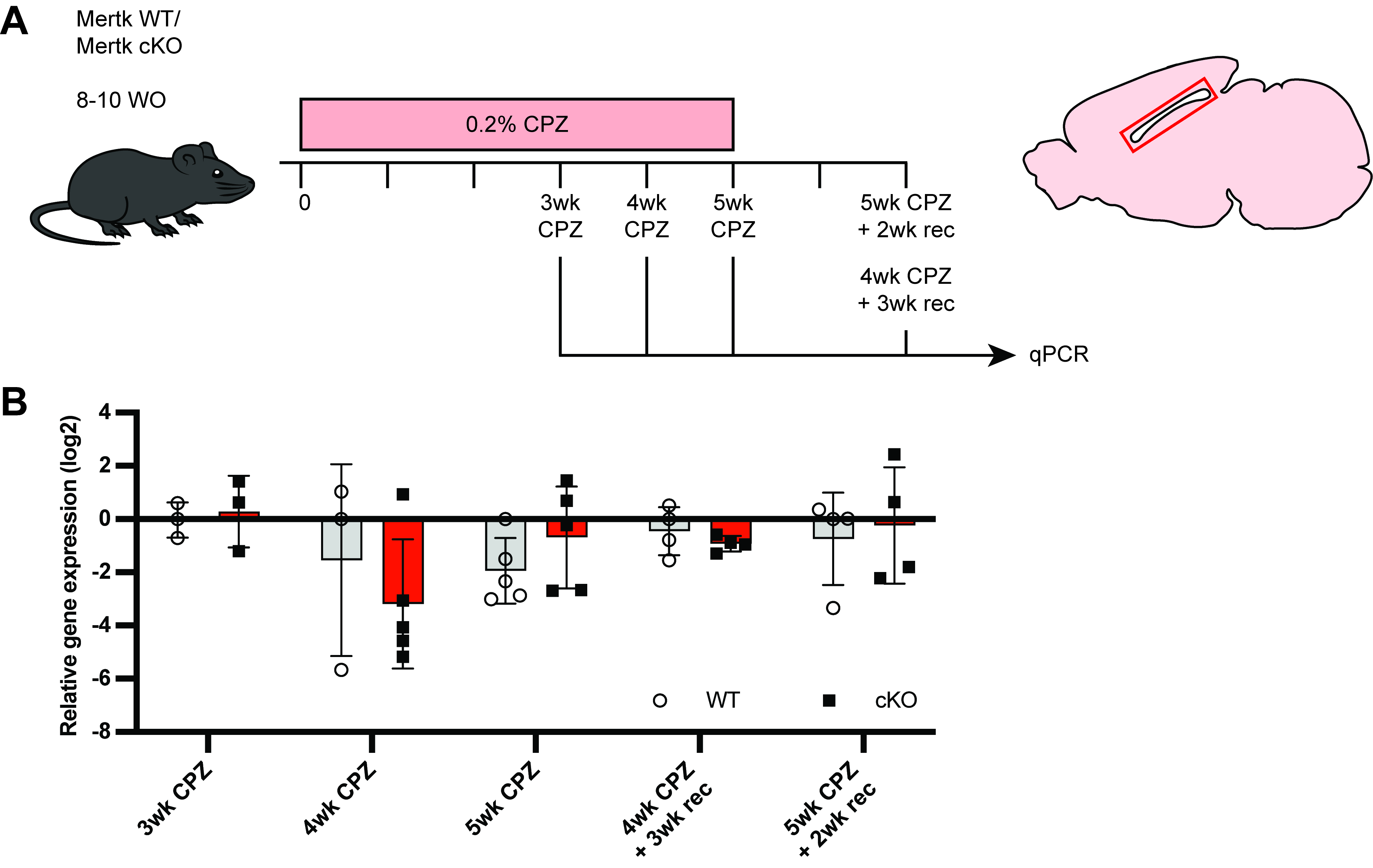

Supplement: Supplementary file 5 — Additional file 5: Figure S5. Ifnγ gene expression is not altered by loss of microglial Mertk during cuprizone-mediated demyelination or subsequent remyelination. A Demyelination was induced in Mertk WT and cKO using cuprizone (0.2% w/w). Mice were collected at demyelination time-points: 3wk CPZ, 4wk CPZ and 5wk CPZ. After cuprizone-challenge, cohorts of mice were returned to standard chow for tissue collection at remyelination time-points: 4wk CPZ + 2wk rec and 5wk CPZ + 2wk rec. Corpus callosum tissue was dissected and processed for RNA extraction and qPCR. B Dissected corpus callosum tissue from cKO and WT animals displayed no differences in Ifng gene expression gene expression at any time-point (P > 0.05). Relative gene expression presented on log2 scale. n = 3–5 biological replicates. All data represent mean ± SD. Data from 3 to 5 weeks CPZ time-points analysed by unpaired t-tests. Data from 2–3 weeks recovery time-points analysed by Welch’s t test. [file 12974_2023_2921_MOESM5_ESM.tif]
