# Supplementary material for: Mertk-expressing microglia influence oligodendrogenesis and myelin modelling in the CNS
Source: J Neuroinflammation. 2023 Nov 6;20:253. doi: 10.1186/s12974-023-02921-8 (PMC10626688; doi:10.1186/s12974-023-02921-8)
Supplement: Supplementary file 6 — Additional file 6. Table S1. Test statistic and confidence intervals of statistical analyses. [file 12974_2023_2921_MOESM6_ESM.pdf]

Figure	Panel	Main test	Main test statistics summary	Pre-planned multiple comparison test	Multiple comparison test statistics summary
Figure S5	B	Student's t-test, unpaired, 2-way	P=0.2739 t=1.204 95% CI = -14.26 to 41.90		
Figure S5	C	Student's t-test, unpaired, 2-way	Fgf2: P=0.3702 t=2.923 95% CI = -0.2930 to 0.6770 Pdgfra: P=0.3837 t=0.9396 95% CI = -0.2970 to 0.6674 Lif: P=0.5402 t=0.6492 95% CI = -0.3045 to 0.5244 Gas6: P=0.3629 t=0.9844 95% CI = -0.3048 to 0.7150 Igf1: P=0.8232 t=0.2335 95% CI = -0.5555 to 0.4587. Lgals3: P=0.5456 t=0.6403 95% CI = -0.3382 to 0.5779 Spp1: P=0.3066 t=0.1.112 95% CI = -0.7084 to 0.2656 Pros1: P=0.8550 t=0.1908 95% CI = -0.4330 to 0.5063 Gpnmb: P=0.8437 t=0.2059 95% CI = -0.4766 to 0.5642		
Figure 2	B	Two way ANOVA	Interaction: F(2,15) = 0.5238 Age: (2, 15) = 39.41 Genotype: (F1, 15) = 0.002032		
Figure 2	B			WT-cKO P28	P=0.4033; 95% CI = -115291 to 271256
Figure 2	B			WT-cKO young adult	P=0.7361; 95% CI = -224404 to 162143
Figure 2	B			WT-cKO Aged	P=0.6672; 95% CI = -233045 to 153502
Figure 2	C	Simple linear regression	WT: P<0.0001, F=116, Y = 0.03821*X + 0.07445. cKO:P<0.0001, F=100.9, Y = 0.03533*X + 0.07847		
Figure 2	C			WT-cKO: Test for difference in slope and elevation	Slope: F=0.3333, P=0.5636; Elevation: F=2.537, P=0.1115
Figure 2	D	Simple linear regression	WT: P<0.0001, F=371.4, Y = 0.05857*X + 0.09789 cKO:P<0.0001, F=248.4, Y = 0.07190*X + 0.08962		
Figure 2	D			WT-cKO: Test for difference in slope and elevation	Slope: F=6.233, P=0.0126
Figure 2	E	Simple linear regression	WT: P=0.0005, F=12.26, Y = 0.01555*X + 0.08486 cKO:P<0.0001, F=103.6, Y = 0.03583*X + 0.06782		
Figure 2	E			WT-cKO: Test for difference in slope and elevation	Slope: F=12.70, P=0.0004
Figure 2	G	Fisher's exact test (two-sided)	P=0.0016		
Figure 2	H	Two way ANOVA	Interaction: F(1, 10) = 5.330 Postnatal day: (F1,10) = 1590 Genotype: (F1, 10) = 15.54		
Figure 2	H			WT-cKO P7	P=0.0013; 95% CI = 0.3200 to 0.9706
Figure 2	H			WT-cKO P15	P=0.2751; 95% CI = -0.1567 to 0.4938
Figure 2	I	Two way ANOVA	Interaction: F(1, 10) = 2.459 Postnatal day: (F1,10) = 1503 Genotype: (F1, 10) = 5.641		
Figure 2	I			WT-cKO P7	P=0.0192; 95% CI = 0.05428 to 0.4864
Figure 2	I			WT-cKO P15	P=0.5812; 95% CI = -0.1608 to 0.2714
Figure 6	B	Student's t-test, unpaired, 2-way	t=0.09813 95% CI = -30.45 to 28.21		
Figure 6	C	Student's t-test, unpaired, 2-way	t=0.5063 95% CI = -34.35 to 51.20		
Figure 6	D	Student's t-test, unpaired, 2-way	t=0.07467 95% CI = -259.9 to 275.4		
Figure 5	C	Two way ANOVA	Interaction: F(1, 11) = 1.042 Weeks cuprizone: (F1,11) = 0.2217 Genotype: (F1, 11) = 0.08844		
Figure 5	D	Simple linear regression	WT: P=0.0009, F=11.47, Y = 0.02429*X + 0.06186 cKO:P=0.0012, F=10.93, Y = 0.02901*X + 0.06037		
Figure 5	D			WT-cKO: Test for difference in slope and elevation	Slope: F=0.1802, P=0.6715; Elevation: F=0.1146, P=0.7352

Figure 5	E	Simple linear regression	WT: P<0.0001, F=58.84, Y = 0.06829*X + 0.03939 cKO:P<0.0001, F=22.01, Y = 0.03657*X + 0.05313		
Figure 5	E			WT-cKO: Test for difference in slope and elevation	Slope: F=6.854, P=0.0094
Figure 6	B	Two way ANOVA	Interaction: F(1, 8) = 0.0157 Weeks cuprizone: (F1,8) = 101 Genotype: (F1, 8) = 0.004667		
Figure 6	B			WT-cKO 3 weeks cuprizone	P=0.9688; 95% CI =-1830 to 1895
Figure 6	B			WT-cKO 5 weeks cuprizone	P=0.8944; 95% CI = -1973 to 1752
Figure 6	C	Two way ANOVA	Interaction: F(1, 8) = 10.59 Weeks cuprizone: (F1,8) = 345 Genotype: (F1, 8) = 5.353		
Figure 6	C			WT-cKO 3 weeks cuprizone	P=0.5248; 95% CI = -51.49 to 93.22
Figure 6	C			WT-cKO 5 weeks cuprizone	P=0.0043; 95% CI = -195.9 to -51.18
Figure 6	D	Two way ANOVA	Interaction: F(1, 8) = 0.02915 Weeks cuprizone: (F1, 8) = 5.7 Genotype: (F1, 8) = 0.4458		
Figure 6	D			WT-cKO 3 weeks cuprizone	P=0.7344; 95% CI = -527.7 to 717.4
Figure 6	D			WT-cKO 5 weeks cuprizone	P=0.5696; 95% CI = -462.5 to 782.6
Figure 7	B	Two way ANOVA	Interaction: F(1, 10) = 0..6214 Treatment: (F1, 10) = 0.2465 Genotype: (F1, 10) = 368.6		
Figure 7	B			WT-cKO No treatment	P<0.0001; 95% CI = 15.20 to 22.14
Figure 7	B			WT-cKO rhGas6	P<0.0001; 95% CI = 13.73 to 20.66
Figure 7	C	Two way ANOVA	Interaction: F(1, 10) = 0.4763 Treatment: (F1, 10) = 0.8766 Genotype: (F1, 10) = 8.733		
Figure 7	C			WT-cKO No treatment	P=0.2609; 95% CI = -0.3412 to 1.408
Figure 7	C			WT-cKO rhGas6	P=0.0543; 95% CI = -0.01621 to 1.733
Figure 7	D	Two way ANOVA	Interaction: F(1, 10) = 0.4724 Treatment: (F1, 10) = 0.2204 Genotype: (F1, 10) = 28.94		
Figure 7	D			WT-cKO No treatment	P=0.0155; 95% CI = 12137 to 104357
Figure 7	D			WT-cKO rhGas6	P=0.0032; 95% CI = 29203 to 121423
Figure 8	C	Two way ANOVA	Interaction: F(1, 12) = 0.3346 Weeks recovery: (F1, 12) = 0.7609 Genotype: (F1, 12) = 0.8944		
Figure 8	D	Simple linear regression	WT: P=0.0054, F=7.857, Y = 0.01455*X + 0.07936 cKO:P=0.0006, F=12.10, Y = 0.01786*X + 0.07042		
Figure 8	D			WT-cKO: Test for difference in slope and elevation	Slope: F=0.2056, P=0.6504; Elevation: F=12.96, P=0.0003
Figure 8	E	Simple linear regression	WT: P<0.0001, F=30.17, Y = 0.02543*X + 0.08612 cKO:P<0.0001, F=17.82, Y = 0.02133*X + 0.08535		
Figure 8	E			WT-cKO: Test for difference in slope and elevation	Slope: F=0.3606, P=0.5484; Elevation: F=2.307, P=0.1292
Figure S4	B	Two way ANOVA	Interaction: F(1, 12) = 0.6024 Weeks recovery: (F1, 12) = 16.13 Genotype: (F1, 12) = 3.780		
Figure S4	B			WT-cKO 2 weeks recovery	P=0.0784; 95% CI =--955.9 to 59.46
Figure S4	B			WT-cKO 4 weeks recovery	P=0.4249; 95% CI = -700.1 to 315.2
Figure S4	C	Two way ANOVA	Interaction: F(1, 12) = 0.1874 Weeks recovery: (F1, 12) = 2.844 Genotype: (F1, 12) = 3.370		
Figure S4	D	Two way ANOVA	Interaction: F(1, 12) = 1.186 Weeks recovery: (F1, 12) = 1.111 Genotype: (F1, 12) = 0.6467		
Figure 9	C	Student's t-test, unpaired, 2-way	D10: P=0.0144 t=2.694 95% CI = 0.8416 to 6.704 R3: P=0.0073 t=3.005 95% CI = 1.985 to 11.10		
Figure 9	E	Student's t-test, unpaired, 2-way	D10: P=0.9339 t=0.08406 95% CI = -4.799 to 5.199 R3: P=0.0017 t=3.675 95% CI = -7.073 to -1.927		
